# A Novel Monoallelic *ALG5* Variant Causing Late-Onset ADPKD and Tubulointerstitial Fibrosis

**DOI:** 10.1016/j.ekir.2024.04.031

**Published:** 2024-04-15

**Authors:** Elhussein A.E. Elhassan, Tereza Kmochová, Katherine A. Benson, Neil K. Fennelly, Veronika Barešová, Kendrah Kidd, Brendan Doyle, Anthony Dorman, Martina M. Morrin, Niamh C. Kyne, Petr Vyleťal, Hana Hartmannová, Kateřina Hodaňová, Jana Sovová, Dita Mušálková, Alena Vrbacká, Anna Přistoupilová, Jan Živný, Klára Svojšová, Martin Radina, Viktor Stránecký, Dmitry Loginov, Petr Pompach, Petr Novák, Zdislava Vaníčková, Hana Hansíková, Silvie Rajnochová-Bloudíčková, Ondřej Viklický, Helena Hůlková, Gianpiero L. Cavalleri, Aleš Hnízda, Anthony J. Bleyer, Stanislav Kmoch, Peter J. Conlon, Martina Živná

**Affiliations:** 1 Department of Nephrology and Transplantation, Beaumont Hospital, Dublin, Ireland; 2 Department of Medicine, Royal College of Surgeons in Ireland, Dublin, Ireland; 3 Research Unit for Rare Diseases, Department of Pediatrics and Inherited Metabolic Disorders, First Faculty of Medicine, Charles University in Prague, Prague, Czech Republic; 4 School of Pharmacy and Biomolecular Sciences, Royal College of Surgeons, Dublin, Ireland; 5 Department of Pathology, Beaumont Hospital, Dublin, Ireland; 6 Section on Nephrology, Wake Forest School of Medicine, Winston-Salem, North Carolina, USA; 7 Department of Radiology, Beaumont Hospital and Royal College of Surgeons in Ireland, Dublin, Ireland; 8 Institute of Microbiology, The Czech Academy of Sciences, Vestec, Czech Republic; 9 Institute of Medical Biochemistry and Laboratory Diagnostics of the General University Hospital and of The First Faculty of medicine of Charles University in Prague, Prague, Czech Republic; 10 Department of Pediatrics and Inherited Metabolic Disorders, First Faculty of Medicine, Charles University in Prague, Prague, Czech Republic; 11 Department of Nephrology, Transplant Center, Institute for Clinical and Experimental Medicine, Prague, Czech Republic

**Keywords:** ALG5, autosomal-dominant polycystic kidney disease, autosomal dominant tubulo-interstitial kidney disease, Golgi apparatus, N-Linked protein glycosylation, UMOD

## Abstract

**Introduction:**

Monoallelic variants in the *ALG5* gene encoding asparagine-linked glycosylation protein 5 homolog (ALG5) have been recently shown to disrupt polycystin-1 (PC1) maturation and trafficking via underglycosylation, causing an autosomal dominant polycystic kidney disease-like (ADPKD-like) phenotype and interstitial fibrosis. In this report, we present clinical, genetic, histopathologic, and protein structure and functional correlates of a new ALG5 variant, p.R79W, that we identified in 2 distant genetically related Irish families displaying an atypical late-onset ADPKD phenotype combined with tubulointerstitial damage.

**Methods:**

Whole exome and targeted sequencing were used for segregation analysis of available relatives. This was followed by immunohistochemistry examinations of kidney biopsies, and targeted (UMOD, MUC1) and untargeted plasma proteome and N-glycomic studies.

**Results:**

We identified a monoallelic *ALG5* variant [GRCh37 (NM_013338.5): g.37569565G>A, c.235C>T; p.R79W] that cosegregates in 23 individuals, of whom 18 were clinically affected. We detected abnormal localization of ALG5 in the Golgi apparatus of renal tubular cells in patients’ kidney specimens. Further, we detected the pathological accumulation of uromodulin, an N-glycosylated glycosylphosphatidylinositol (GPI)-anchored protein, in the endoplasmic reticulum (ER), but not mucin-1, an O- and N-glycosylated protein. Biochemical investigation revealed decreased plasma and urinary uromodulin levels in clinically affected individuals. Proteomic and glycoproteomic profiling revealed the dysregulation of chronic kidney disease (CKD)-associated proteins.

**Conclusion:**

ALG5 dysfunction adversely affects maturation and trafficking of N-glycosylated and GPI anchored protein uromodulin, leading to structural and functional changes in the kidney. Our findings confirm ALG5 as a cause of late-onset ADPKD and provide additional insight into the molecular mechanisms of ADPKD-*ALG5*.

## Graphical abstract

**Figure:**
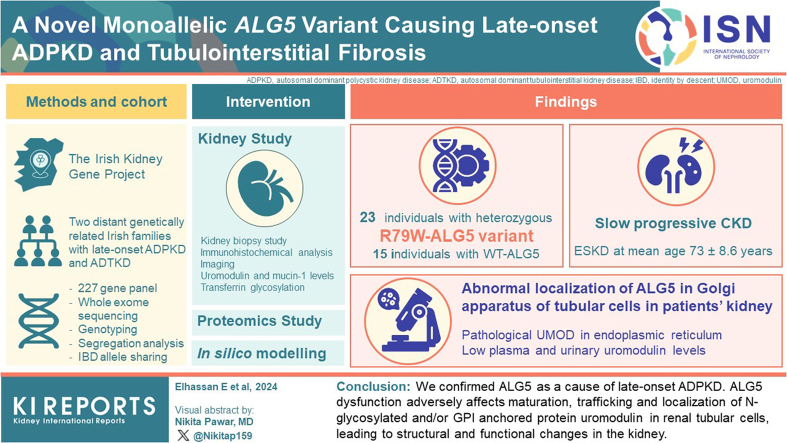

ADPKD is a ciliopathy distinguished by its phenotypic and genetic heterogeneity.^1^, ^2^, ^3^ Pathogenic variants in *PKD1* and *PKD2* genes, which code for PC1 and polycystin-2, respectively, are the most common genetic causes of ADPKD, accounting for approximately 93% of cases of ADPKD.^4^ The median age of end-stage kidney disease (ESKD) is 58 years for *PKD1* pathogenic variants versus 79 years for *PKD2* pathogenic variants.^3^ Other genetic lesions have been identified that result in a cystic renal phenotype, including monoallelic variants in *GANAB*, *DNAJB11*, *ALG5, ALG8*, *ALG9*, and *IFT140*.^5^

A recent study identified 5 monoallelic pathogenic variants in the *ALG5* gene encoding ALG5 protein as a cause of ADPKD, with a phenotype including nonenlarged cystic kidneys, minimal liver cysts, and late-onset CKD progression to ESKD.^6^ *In vitro* studies identified that ALG5 dysfunction results in reduced efficiency of protein glycosylation, compromising the maturation and trafficking of PC1.^6^

In this study, we describe 2 distant genetically related multiplex Irish families with nonenlarged polycystic kidneys and tubulointerstitial fibrosis caused by a novel monoallelic *ALG5* variant and characterize its clinical, histopathologic, protein structural, and functional correlates. In addition, we show abnormalities in trafficking of uromodulin, another N-linked glycoprotein involved in the development of CKD. Our findings confirm *ALG5* as a cause of ADPKD and provide additional insight into the molecular mechanisms of ADPKD-*ALG5*.

## Methods

### Subjects and Study Approval

A nonconsanguineous kindred was referred to our genetic service at the Irish Kidney Gene Project. All individuals provided informed consent. The study was approved by the Ethical Committee of Beaumont Hospital (REC 19/28). Approval for the release of biopsy material was also obtained.

Clinical information and imaging findings were obtained through chart review or clinic visits. Hypertension was defined by blood pressure above 140/90 mm Hg and/or the prescription of antihypertensive medications. CKD was defined as an estimated glomerular filtration rate (eGFR) less than 60 ml/min per 1.73 m^2^ for 3 months or longer, according to the CKD-Epidemiology Collaboration 2021 equation and graded according to the Kidney Disease Improving Global Outcomes Guidelines.^7^^,^^8^ Screening for proteinuria was assessed using the spot urine protein-to-creatinine ratio (mg/mmol). The commencement of kidney replacement therapy or preemptive kidney transplant was defined as ESKD. Ultrasound was used to assess kidney and liver size, echogenicity, and the presence of cysts. If unavailable, previous imaging studies were examined. An individual was considered to be clinically affected if 1 of the following clinical characteristics was present: multiple kidney cysts, nonenlarged kidneys with nephronophthisis-like histological characteristics, or CKD (for method details, see Supplementary Methods).S17

### Genetic Analysis

Genomic DNA was extracted from blood using standard procedures. Genetic testing included Sanger sequencing of *UMOD*,^9^ and *REN*,^10^ targeted genotyping^11^ and long-read sequencing of *MUC1*,^12^ 227-gene panel,^13^^,^^14^ whole-exome sequencing, and targeted *ALG5* variant genotyping. Data analysis, variant prioritization, and targeted genotyping were performed as previously described.^15^ PLINK 1.9 was used to estimate genome-wide identity-by-descent–sharing coefficients between members of both families (*n* = 4) to determine whether the *ALG5* variant is of a common descent^16^ (Supplementary Table S1).S18–S25

### *In-Silico* Analysis

For structural mapping, the AlphaFold model of human dolichyl-phosphate beta-glucosyltransferase activity was used (i.e., AF-Q9Y673-F1). Regions involved in enzymatic catalysis and position of the substrates were determined by a superposition with archeal dolichyl-phosphate mannose synthase crystal structures as reported previously.^17^ Structural models were visualized using Pymol and ChimeraX.S26

### Histopathological Staining

Formaldehyde-fixed paraffin-embedded kidney biopsies were available in 3 affected subjects. Staining with Masson's trichrome, hematoxylin-eosin and silver were performed according to manufacturer’s protocols. Antisera against IgG, IgM, IgA, fibrin, C3, C4, and kappa and lambda light chains, were utilized to detect deposition via indirect immunofluorescence. Tubulointerstitial lesions, such as tubulointerstitial fibrosis and tubular atrophy, was assessed semiquantitatively via visual inspection using Masson's Trichrome-stained kidney sections (5 μm) to determine the percentage of interstitial fibrosis present in the cortical specimens of kidney biopsies.

### Immunohistochemical and Immunofluorescence Analysis of Human Kidney Biopsy

The paraffin sections were stained after deparaffination, hydration, sodium citrate pretreatment (pH = 6), and standard blocking procedures. Immunodetection of ALG5 was achieved with antihuman ALG5 antibody (Novusbio, NBP2-92371) diluted 1:100 in PBS followed by detection with Dako EnVision+TM Peroxidase Rabbit Kit (Dako, Glostrup, Denmark).

For parallel immunodetection of either ALG5, UMOD, or MUC1 with ER, ER-Golgi intermediate compartment, Golgi apparatus, or plasma membrane markers, the following antibodies were used: ALG5 with polyclonal rabbit anti-ALG5 antibody (Novus Biologicals) diluted 1:200; UMOD with polyclonal sheep anti-THP/UMOD antibody (Tamm-Horsfall Glycoprotein antibody, My BioSource) diluted 1:300; MUC1 with monoclonal Mouse antiHuman Epithelial Membrane Antigen antibody (Dako, Glostrup, Denmark) diluted 1 : 100; ER with monoclonal mouse anti-PDI antibody (ENZO Life Sciences) diluted 1:50 or rabbit anti-SEC61A (Abcam #14379] diluted 1 : 300; ER-Golgi intermediate compartment with monoclonal mouse anti-LMAN1 antibody (Thermo Fisher Scientific) diluted 1:100; Golgi apparatus with monoclonal mouse anti-58K antibody (Abcam) diluted 1:50 and plasma membrane with rabbit antipan Cadherin (Thermo Fisher Scientific) diluted 1:50 in 5% BSA in PBS. Detection of bound primary antibodies was achieved using Donkey anti-Mouse IgG Alexa Fluor 647, Donkey anti-Rabbit IgG Alexa Fluor 488 and Donkey anti-Sheep IgG Alexa Fluor 555 secondary antibodies (Thermo Fischer Scientific) diluted 1:500 in 5% BSA in PBS. For method details, see Supplementary Methods.

### Image Acquisition and Analysis

XYZ images were sampled according to the Nyquist criterion using a Leica SP8X laser scanning confocal microscope. Images were restored using a classic maximum likelihood restoration algorithm in the Huygens Professional Software (SVI, Hilversum, The Netherlands).^18^ The Pearson coefficients of the colocalization and the colocalization maps employing single pixel overlap coefficient values ranging from 0 to 1 were created in the Huygens Professional Software.^19^ The resulting overlap coefficient values are presented as the pseudo color, which scale is shown in corresponding lookup tables.S27S28

### Western Blot Analysis of Urinary Uromodulin

Aliquots of spot urines were normalized to urinary creatinine concentration. Excreted uromodulin was detected by western blot using polyclonal sheep anti-THP/UMOD antibody (Tamm-Horsfall Glycoprotein antibody, My BioSource) diluted 1:5000 and Donkey anti-Sheep IgG (H+L)–Peroxidase antibody (Thermo Fisher Scientific) diluted 1:10 000 in 1x PBS-Tween (0.1 %) with 1% BSA (1 h each). Membranes were developed in 1:1 mixture of Clarity Western ECL Substrate (Bio-Rad) solutions. Chemiluminescent signal was recorded by ChemiDoc MP Imaging System (Bio-Rad). For method details, see Supplementary Methods.

### Plasma Measurements

Plasma uromodulin concentration was determined by the Uromodulin Human ELISA kit (Biovendor, Czech Republic). Plates were read in SLT Spectra plate reader (SLT Labinstruments GmbH, Austria). Measured data were processed by KIM Immunochemical Processing software (Daniel Kittrich, Czech Republic). Plasma CA15-3 (mucin 1) concentration was determined as previously described.^20^^,^S29

### Western Blot Analysis of Plasma Transferrin

Electrophoresis and western blot of plasma transferrin were performed according to Seta *et al.*^21^ with minor modifications. For method details, see Supplementary Methods.S30

### Proteomic and Glycoproteomic Profiles of Plasma

Plasma protein concentration was determined using a BCA Protein Assay Kit (ThermoScientific). From each sample, an aliquot equivalent to 100 μg of protein was taken and divided into 2 parts. One part was subjected directly to the proteomic analysis, whereas the other one underwent an albumin depletion protocol,^22^ to enrich glycoprotein content.

Proteomic analysis was carried out using a sample aliquot containing 20 μg of protein.

After the protein digestion and desalting,^23^ the sample was dried, resuspended, and analyzed. The analysis of the peptides was performed using the Vanquish liquid chromatography system (ThermoScientific), which was coupled to the timsToF SCP mass spectrometer equipped with Captive spray (Bruker Daltonics). The mass spectrometer operated in a positive data-dependent mode. Proteins were identified using MaxQuant software (version 1.6.17),^24^ and the Homo sapiens database from Uniprot using the Andromeda search engine.^25^

For label-free quantification, the MaxLFQ algorithm^26^ integrated into MaxQuant was utilized. A minimum ratio count of 2 was set to ensure robust quantification. Subsequent data analysis was performed using Perseus software (version 2.0.70).^24^ Label-free quantification intensity values were log2-transformed and normalized using Z-score. Statistical analyses were carried out using algorithms integrated into Perseus.

The raw data were deposited to a ProteomeXchange Consortium via a PRIDE^27^ partner repository with a data set identifier.S31–S37

### Statistical Analysis

Patient characteristics and genetic diagnosis were collected, and data were presented descriptively; continuous variables were expressed as mean ± SD or median (interquartile range), whereas categorical variables were expressed in frequencies or percentages. The difference in means between subgroups was determined using a 2-sample Student t-test, with a 2-tailed *P*-value of less than 0.05 indicating statistical significance. Kaplan-Meier curves with log-rank testing were used to evaluate progression to ESKD. Statistical analyses were performed using STATA SE 16 (StataCorp, College Station, TX).

## Results

### Genetic Investigations Identified a Pathogenic *ALG5* Variant in 2 Families

**Figure 1:**
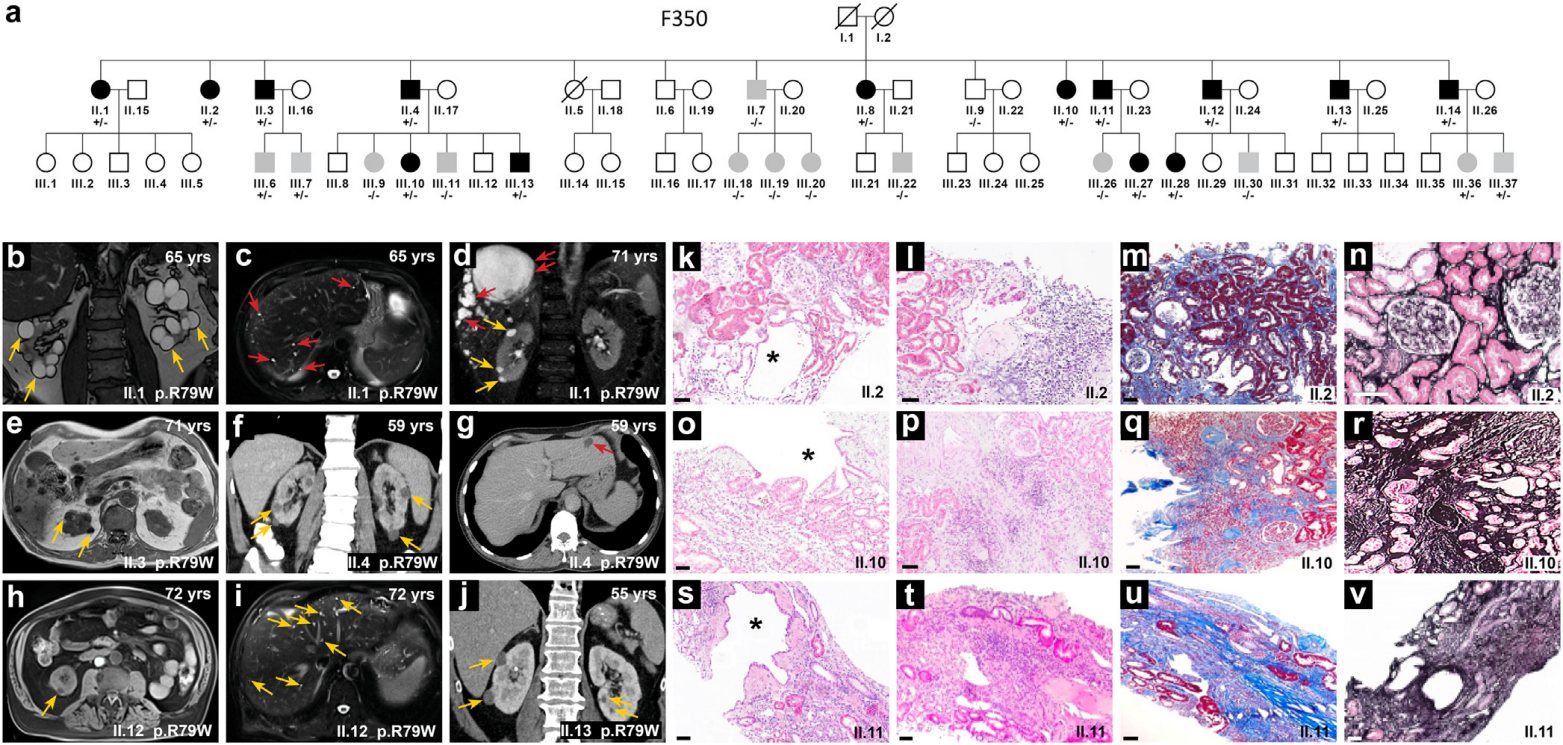
Family F350. (a) The F350 pedigree shows a family with autosomal dominant atypical polycystic kidney disease and a slow decline in glomerular filtration rate (GFR). Clinically affected individuals (black symbols) had bilateral kidney cysts, reduced GFR below 60 ml/min per 1.73 m^2^ or nonenlarged kidneys with nephronophthisis-like histology. Gray indicates young carriers of the variant with no established clinical diagnosis yet. White indicates clinically unaffected individuals. Out of the 28 individuals evaluated in this family, 18 were determined as genetically affected individuals with the heterozygous missense variant R79W-ALG5 (+/−), whereas 10 individuals were genetically unaffected (−/−). A (+) sign indicates the presence of the R79W-ALG5 variant, and a (−) sign indicates the presence of the wild-type WT-ALG5 in a genotyped individual. (Bottom - Left) Radiologic imaging of genetically affected members of F350 (b–j). Yellow arrows denote kidney cysts, whereas red arrows indicate cysts in the liver. (b and c) T2-weighted magnetic resonance imaging (MRI) of 65-year-old patient II.1 demonstrates bilateral multiple scattered kidney and liver cysts. (d) At age 64, II.3 underwent an MRI scan that revealed nonenlarged cystic kidney and high-burden hepatic simple cysts (>20 cysts, with the largest measuring 11.6 cm in the right lobe - red arrows;). He underwent liver cysts fenestration at 70 years of age as the result of ongoing pain. An MRI of the liver performed at the age of 71 years (e) confirms the presence of severe polycystic liver disease. (f and g) A CT scan of II. 4's kidneys and liver at age 59 showed multiple subcentimeter-sized kidney cysts and a solitary liver cyst in segment 2. Similar to II.3, a 72-year-old MRI scan of II.12 reveals scattered kidney cysts (1 cyst is displayed in h) and (i) numerous liver cysts, whereas (j) a CT scan of II.13 reveals only a few kidney cysts. (Bottom - Right) (k, o, and s) Kidney biopsy slides staining of 3 genetically affected individuals from F350. At 52, 51, and 54 years of age, subjects II.2, II.10, and II.11, respectively, underwent clinically indicated kidney biopsies; their corresponding estimated glomerular filtration rate at the time of the biopsies were 37, 32, and 54 ml/min per 1.73 m^2^. Hematoxylin and eosin (H&E) stained section showing a central cystically dilated tubule in all patients marked with an asterisk (∗). (Images l, p, and t) Also, a variable degree of inflammatory infiltrate within the tubulointerstitial compartment has been identified–using H&E stain. (m, q, and u) Masson-Trichrome and (n, r, and v) Silver stained sections illustrating variable degrees of glomerulosclerosis, interstitial fibrosis, and tubular atrophy. All biopsies captured at 10× magnification with scale bars represent 100 μm. CT, computed tomography; MRI, magnetic resonance imaging.

**Figure 2:**
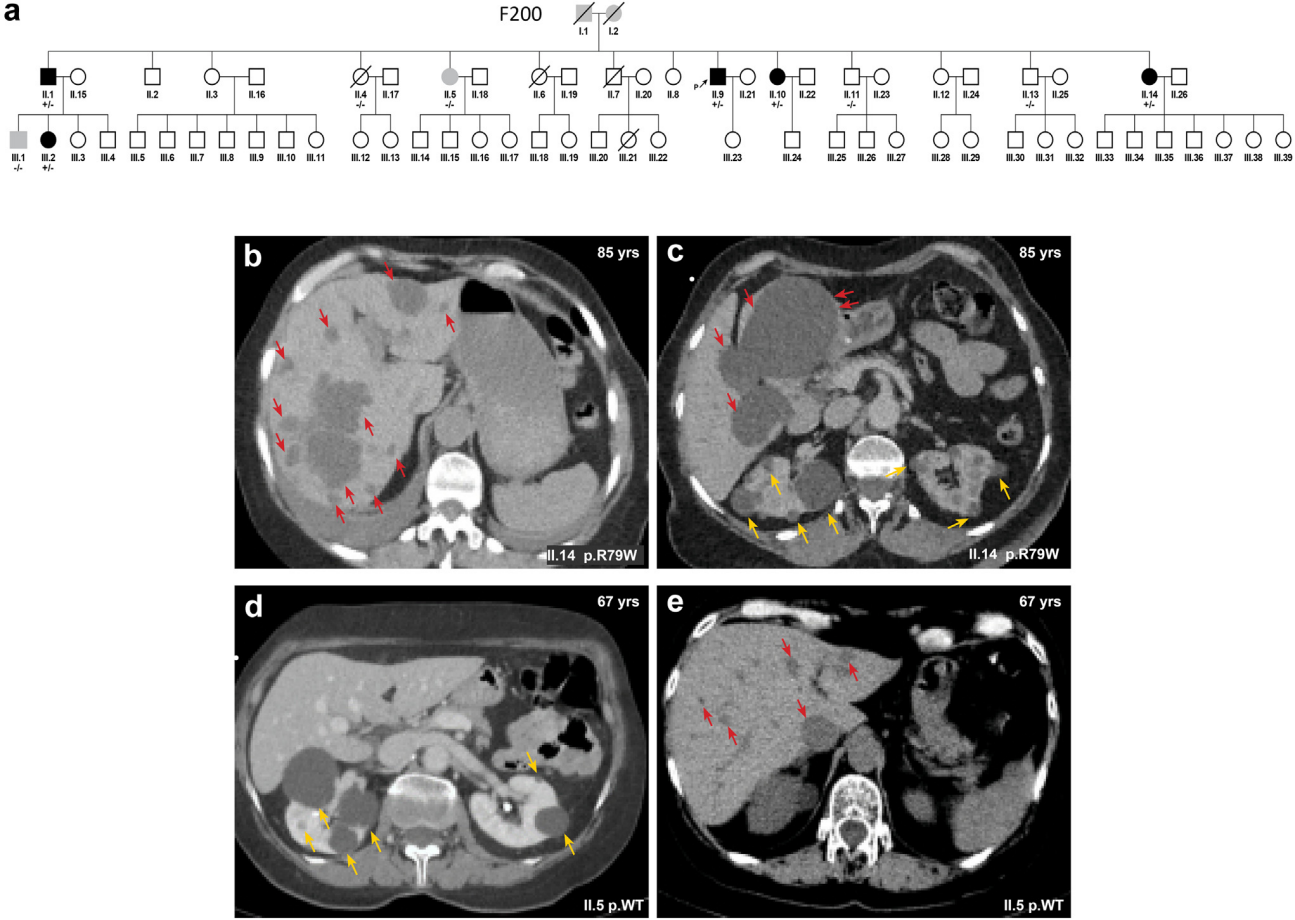
Family F200. (a) The pedigree of F200 shows 5 clinically affected members (black symbols). Gray indicates clinically unclear individuals and white indicates clinically unaffected individuals. A (+/−) sign indicates the presence of the heterozygous missense variant R79W-ALG5 and a (−/−) sign indicates the presence of the wild-type WT-ALG5. A (+) sign indicates the presence of the R79W-ALG5 variant, and a (−) sign indicates the presence of the wild-type WT-ALG5 in a genotyped individual. Out of the 10 individuals evaluated in this family, 5 were determined as genetically affected individuals with a heterozygous missense variant R79W-ALG5 (+/−), whereas the remaining members were genetically unaffected (−/−). (b and c) CT scan of II.14 at the age of 86 years revealed nonenlarged cystic kidneys (yellow arrows) and severe polycystic liver disease (red arrows). (d and e) Subject II.5 at the age of 67 years, who does not harbor the *ALG5* variant, has a few kidney and scattered liver cysts with estimated glomerular filtration rate 57 ml/min per 1.73 m^2^. Disease-causing variants in *PKD1, PKD2, PRKCSH, ALG9, ALG8, DNAJB11, SEC63,* and *GANAB*, among other monogenic kidney disorders, were analyzed and none were identified, using exome sequencing. Subjects II.4, II.11 and II.13 enjoy a preserved kidney function; eGFR 66 ml/min for subject II.4, 80 ml/min for subject II.11 and 69 ml/min for subject II.13 at age of 76, 77, and 73 years, respectively. None of the subjects had any kidney cysts: Subject II.4 (at age 76 years via CT scan with contrast with slice thickness of 1 mm), subject II.11 (at age 69 years via CT abdomen and pelvis imaging with contrast; slice thickness of 1.5 mm) and subject II.13 (at age 70 years via US abdomen). Subjects II.4 and II.11 have been found to have small simple liver cysts, estimated to be less than approximately 5 to 10 in count. CT, computed tomography; US, ultrasound.

After screening for *PKD1*, *PKD2*, *UMOD, HNF1B,* and *REN* variants (using a 227 kidney gene panel^13^^,^^14^) and reading frame-changing variants in *MUC1* gene (using Illumina and PacBio sequencing^12^^,^^20^), no causative variant was identified in 3 family members of F350 (Figure 1). Using WES in the proband (II.1) and 7 of her affected siblings (II.2, II.3, II.4, II.8, II.10, II.11, and II.12), we identified a unique variant (GRCh37: g.37569565G>A) encoding for a missense variant in *ALG5* (NM_013338.5:c.235C>T; p.R79W) shared with all affected individuals. Results were validated by Sanger sequencing of the mutated exonic fragment. An extended co-segregation analysis was then carried out in family F350, identifying an additional 10 genetically affected individuals [for a total of 18 affected individuals with heterozygous R79W-ALG5 variant and 10 relatives who did not carry the variant (WT-ALG5)] (Figure 1). Searching specifically for *ALG5* variants in other Irish families with an unresolved ADPKD/ADTKD-like phenotype (as defined above), we identified an additional family (F200), previously not known to be related to the index family, carrying the same variant (Figure 2). Using Sanger sequencing and segregation analysis, we genotyped a total of 38 individuals from both families and identified 23 genetically affected individuals and 15 individuals who did not carry the variant. The identity-by-descent estimates infer that both families are part of the same extended family (Supplementary Table S1). Subjects F200_II.5 (aged 67 years [eGFR: 57 ml/min]; Figure 2 Panel D and E) and F350_II.7 (aged 69 years (eGFR: 79 ml/min); figures not shown), neither harboring the heterozygous R79W-ALG5 variant, have a few scattered liver and kidney cysts. Exome sequencing was performed, and disease-causing variants *PKD1, PKD2, PRKCSH, ALG9, ALG8, DNAJB11, SEC63*, and *GANAB*, among other monogenic kidney disorders, were analyzed. No pathogenic variants were identified. F200 subjects II.4, II.11, and II.13 have preserved eGFR, no kidney cysts, and WT-ALG5; however, II.4 and II.11 developed liver cysts at age 76 and 69 years, respectively (Figure 2).

### *In-silico* Prediction of the Structural and Functional Impact of the p.R79W Variant

**Figure 3:**
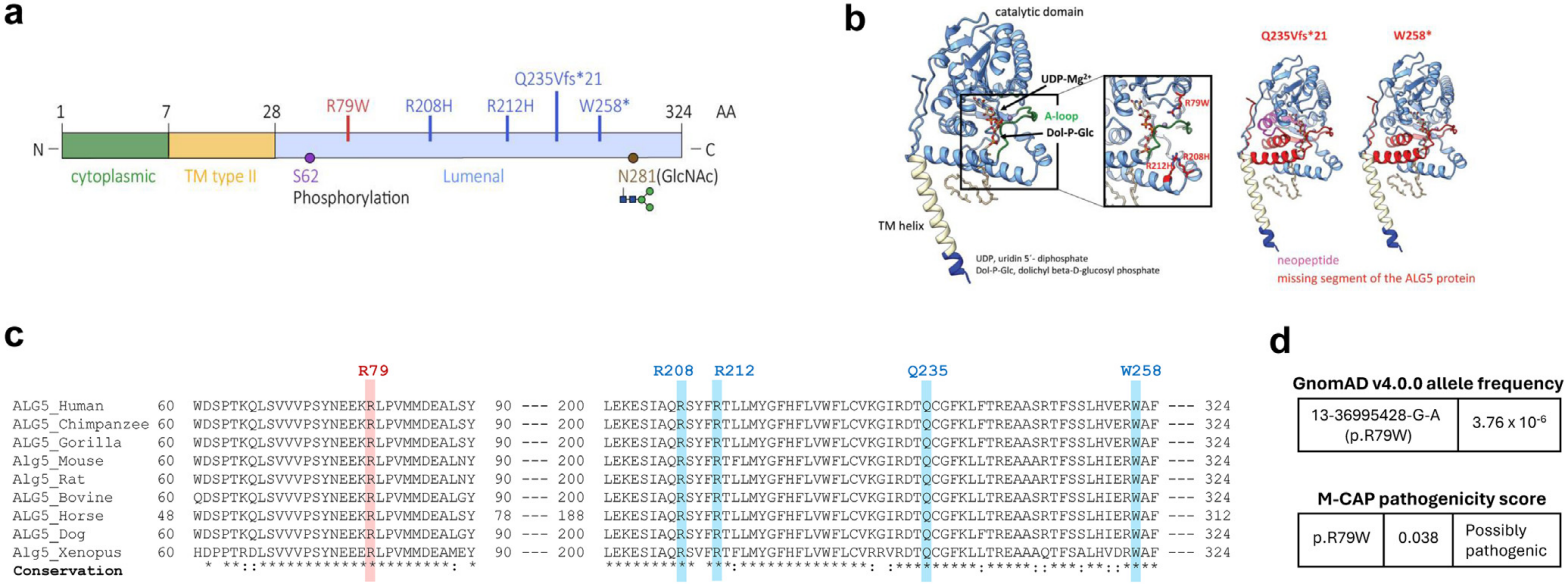
*In Silico* structural mapping of missense variants in ALG5. (a) Diagram of the ALG5 sequence. Novel pathogenic variant identified in this study is shown in red. Previously reported dominant pathogenic variants associated with late-onset ADPKD are shown in blue. (b) Computational structural model of human dolichyl-phosphate beta-glucosyltransferase activity generated by AlphaFold (AF-Q9Y673-F1). Reaction products, dolichyl-phosphate (Dol-P) and Uridine 5'-diphospho (UDP)-alpha-D-glucose (UDP-Glc), are placed at the enzyme active site according to data from the crystal structure of archeal dolichyl-phosphate mannose synthase (see methods). Flexible region of the A-loop governing a dolichyl-phosphate beta-glucosyltransferase activity is highlighted in green. Mutated arginine residues previously reported (R208H, R212H) and the variant from this study (R79W) are shown as red sticks. The last 2 models represent mutants carrying premature stop codons. Missing parts of ALG5 protein and neopeptide in Gln235Valfs∗21 are highlighted by red and magenta, respectively. (c) Amino acid conservation across mutated segments of ALG5 in mammals. Asterisks (∗) indicate amino acid residues that are absolutely conserved and a colon (:) indicates residues with strong conservation across species. (d) Population allele frequency of R79W-ALG5 variant in GnomAD v4.0.0 database and computational prediction by Mendelian Clinically Applicable Pathogenicity Score (M-CAP) of the pathogenicity of missense R79W-ALG5 variant. ADPKD, autosomal dominant polycystic kidney disease.

ALG5 is an ER-resident glycoprotein participating in glucosylation of the oligomannose core in the process of N-linked glycosylation of proteins.^28^ Population variation analysis, sequence alignment, and theoretical AlphaFold-generated model (AF-Q9Y673-F1) revealed that the p.R79W variant was not identified in any reference population databases and is absolutely evolutionarily conserved across species. The other 2 *ALG5* missense substitutions in ADPKD families (p.R208 and p.R212) are similar to p.R79,^6^ in which the variant also replaces an arginine residue in the close proximity of the A-loop of the enzyme that governs dolichyl-phosphate beta-glucosyltransferase activity (Figure 3b and c). The variant p.R79W-ALG5 is classified by the M-CAP pathogenicity classifier^29^ as possibly pathogenic (Figure 3d).

### Clinical Description of the ALG5 Affected Families

**Figure 4:**
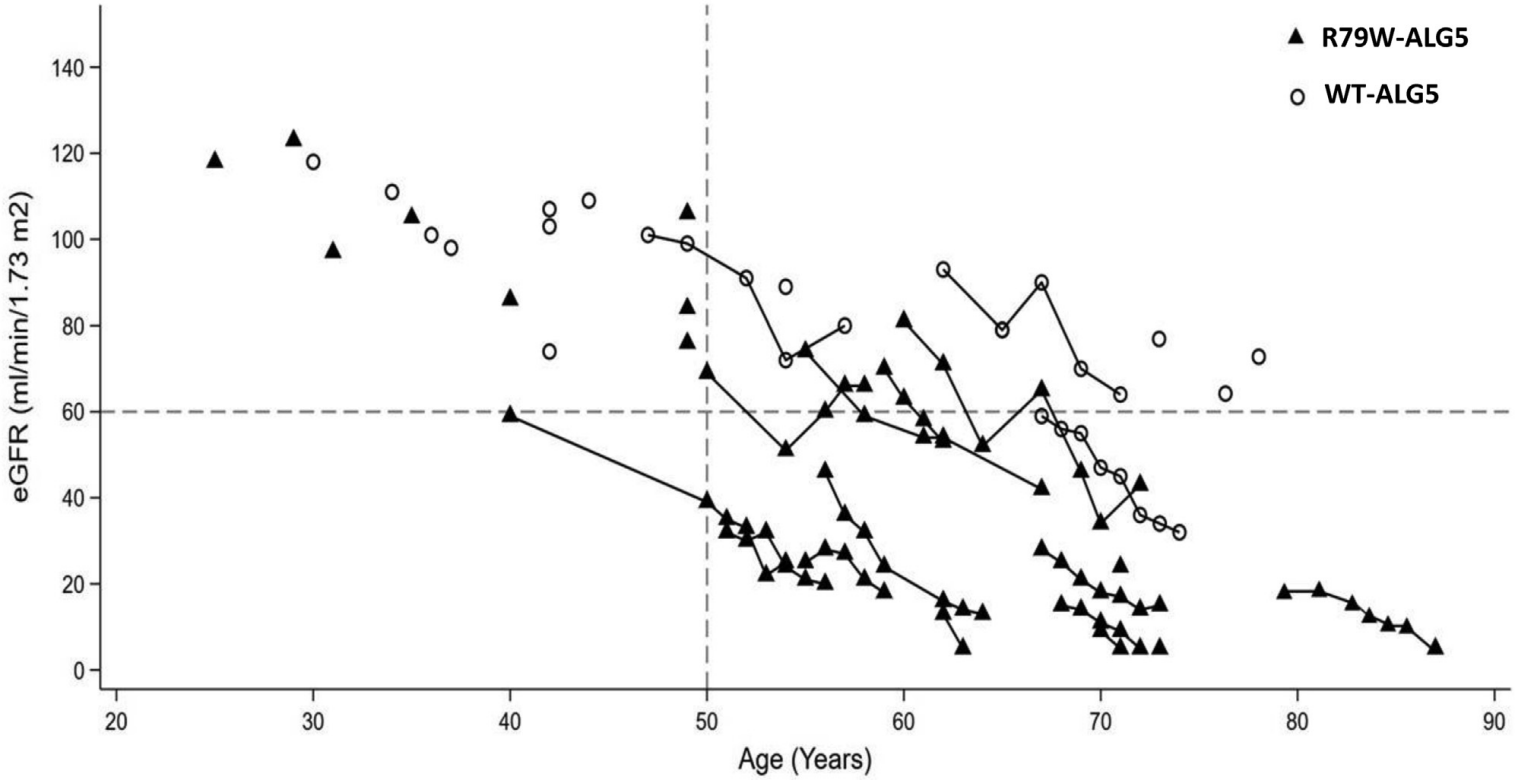
Kidney function in genetically affected individuals. The most recent estimated glomerular filtration rate (eGFR) (ml/min per 1.73 m^2^) versus age in individuals affected with the missense mutation p.R79W in ALG5. Solid triangles indicate genetically affected individuals. Hollow circles indicate genetically unaffected individuals; none of whom developed chronic kidney disease, except on individual F200-II.9 (individual with a history of multiple comorbidities, namely atrial fibrillation, osteoarthritis, recurrent cystitis, microscopic hematuria, and active tobacco use; please refer to the manuscript for details). Most affected individuals developed chronic kidney disease stage 3 (horizontal line) after the age of 50 years (vertical line). Five individuals of varying ages had reached end-stage kidney disease, as indicated by an eGFR value of 5 ml/min per 1.73 m^2^ (Y-axis).

We identified 23 genetically affected individuals with the heterozygous R79W-ALG5 variant and 15 genetically unaffected individuals without the variant (WT-ALG5). Figures 1 and 2 depict the pedigrees of the 2 multiplex nonconsanguineous families with polycystic kidneys, variable cystic liver phenotype, and CKD. Affected individuals developed slowly progressive CKD. None of the affected individuals developed an eGFR < 60 ml/min per 1.73 m^2^ before age of 50 years. Compared to 1 of 15 (6.7%) genetically unaffected individuals, 14 of 23 (60.9%) genetically affected individuals had CKD (*P*-value: 0.002, Fisher Exact). Five patients, all of whom harbored the heterozygous R79W-ALG5, reached ESKD at a mean age of 73 ± 8.6 years (range: 63–87 years), without a significant gender difference (Figure 4 and Supplementary Figure S1A). All but 1 of the affected individuals underwent radiologic evaluation. Eight affected had kidney and liver cysts, 7 only kidney cysts, and 7 no liver or kidney cysts at mean ages of 72.7 ± 12, 55.1 ± 15.8, and 50.2 ± 15.7 years, respectively (Supplementary Figure S1B). Patients with advanced CKD did not have enough displacement of normal kidney tissue with renal cysts to account for the degree of kidney failure, compared with ADPKD,^30^ but kidney cyst burden (total number of kidney cyst) was significantly inversely correlated with eGFR at last follow-up (*r* = −0.539, *P*-value: 0.009). A study of kidney volume in 7 genetically affected individuals found that their kidneys were rather not enlarged, with an average height-adjusted total kidney volume of 156 ± 122 ml/m at age 70.4 ± 9 years, indicating Mayo Class 2B. A polycystic liver phenotype (>20 cysts) was present in 2 individuals. In summary, out of the 23 individuals who were genetically affected, 18 were clinically affected (11 had an eGFR <60 ml/min per 1.73 m^2^ and kidney cysts; 3 had nonenlarged kidneys with nephronophthisis-like histological characteristics; and 4 had kidney cysts and an eGFR >60 ml/min per 1.73 m^2^); the remaining 5 are clinically unaffected so far. The clinical description of affected family members can be found in Table 1.

**Table 1:** Clinical and radiological characterization of the 23 affected members in whom the novel pathogenic R79W-ALG5 variant was confirmed

ID	Sex	eGFR^a^ at last F/U (Age, yr)	uPCR^b^ at last F/U (Age, yr)	HTN (Age)	Kidney Imaging				Liver Imaging	Comorbidities (Age, yr)
					Type	Age, yr	Cysts Description^c^	Diameter (R/L), mm	Cysts	
Pedigree F350 (18 individuals)										
II.1	F	ESKD (71)	47 (71)^d^	Y (55)	MRI	65	>20 cysts bilaterally (largest 34 mm)	65 / 67	∼15 scattered cysts (largest 7 mm)	Diverticulosis (67)
II.2	F	18 (59)	2.5 (59)	N (55)	US	53	No kidney cysts identified	84 / 81	None	None
II.3	M	15 (74)	20 (73)	Y (69)	MRI	71	∼5–10 bilateral cysts (largest 15 mm)	85 / 95	∼30 scattered cysts (largest 24 mm)	Liver cysts fenestration (70 years) for ongoing pain
II.4	M	ESKD (63)	78 (62)^d^	Y (50)	CT	59	∼10 bilateral cysts (largest 31 mm)	88 / 114	1 cyst (16 mm)	IHD (49), Emphysema (55)
II.8	F	66 (57)	NA	N (58)	US	56	16 mm right kidney cyst and ∼5 simple cysts on the left kidney (largest 20 mm)	114 / 97	Unknown	None
II.10	F	25 (55)	11 (55)	N (55)	US	51	∼5 bilateral cysts	86 / 81	Unknown	None
II.11	M	16 (63)	NA	Y (57)	US	63	19 mm right kidney cyst and no cysts on the left kidney	100 / 82	None	None
II.12	M	24 (71)	12 (70)	Y (68)	MRI	72	∼5 right kidney cysts (largest 10 mm) left pelvic kidney with 1 cyst measured 19 mm	108 / 103	10 – 15 cysts (largest 5 mm)	Diverticulosis (65) Metastatic SCLC (71)
II.13	M	59 (61)	NA	N (61)	CT	55	∼15 bilateral cysts (largest 62 mm)	145 / 141	10 – 15 cysts (largest 8 mm)	DM, OSA, cauda equina, cortical nephrocalcinosis
II.14	M	47 (66)	9 (63)	Y (62)	US	66	1 cyst on the right kidney (measured 13 mm) and ∼ 5 left kidney cysts (largest 25 mm)	103 / 111	1 cyst (11 mm)	None
III.6	M	>90 (50)	<15 (49)	N (49)	US	50	No kidney cysts detected	104 / 95	None	liver haemangiomas (50)
III.7	M	78 (46)	6 (45)	N (46)	US	46	No kidney cysts detected	111 / 111	None	None
III.10	F	>90 (40)	15 (40)	N (41)	US	41	1 cyst on the right kidney (measured 23 mm)	99 / 114	None	None
III.13	M	>90 (34)	8 (35)	N (35)	US	35	1 cyst on each kidney (largest 12 mm)	115 / 129	None	None
III.27	F	>90 (31)	NA	N (32)	US	32	No kidney cysts detected	11.2 / 10.4	None	None
III.28	F	84 (50)	1 (49)	N (50)	US	50	∼5 bilateral cysts (largest 10 mm)	10.2 / 10	None	None
III.36	F	>90 (29)	5 (29)	N (29)	US	29	No kidney cysts detected	11.7 / 12.1	None	None
III.37	M	>90 (25)	3 (24)	N (25)	NA	-	Unknown	-	Unknown	None
Pedigree F200 (5 Individuals)										
II.1	M	ESKD (72)	4.2 (68)	Y (60)	US	71	2 cyst on the right kidney (measured 14 mm) and ∼ 6 left kidney cysts (largest 13 mm)	84 / 94	∼5 cysts	NODAT (80), Meniere’s disease (65)
II.9	M	ESKD (73)	NA	Y (NA)	CT	65	∼5-10 bilateral cysts	84 / 81	Unknown	None
II.10	F	44 (72)	NA	NA	US	65	2 cysts in the right kidney	9.4 / 9	∼5 cysts	Diverticulosis (65)
II.14	F	ESKD (87)	37 (86)^d^	Y (82)	CT	86	∼15 bilateral cysts (largest 40 mm)	9.9 / 10	> 30 scattered cysts (largest 95 mm)	None
III.2	F	25 (54)	4.4 (9.1)	Y (50)	US	49	∼5 bilateral cysts (largest 5 mm)	11.1 / 10.3	∼5 cysts	None

CKD-EPI, Chronic Kidney Disease-Epidemiology Collaboration; CT, computed tomography; DM, diabetes mellitus; eGFR, estimated glomerular filtration rate; ESKD, end-stage kidney disease; F, female; F/U, Follow-up; HTN, hypertension; ID, patient identifier; IHD, ischemic heart disease; L, left kidney; M, male; MRI, magnetic resonance imaging; N, no; NA, not available; OSA, obstructive sleep apnea; R, right kidney; uPCR, urine protein-to-creatinine ratio; US: ultrasound; Y, yes.

a Expressed as ml/min per 1.73 m^2^ on the basis of the last data available (CKD-EPI formula).

b Measured in mg/mmol.

c Kidney and liver cysts were described as per available imaging technique. If a particular imaging was not performed, it is called unknown.

d uACR was measured near or after progression into ESKD.

### Histopathological Findings

Although histological features varied across biopsies from the 3 genetically affected individuals, common findings included the presence of cystically dilated tubules and secondary glomerulosclerosis (Figure 1). There was approximately 25%, 25%, and 50% tubulointerstitial fibrosis and tubular atrophy in the 3 biopsies. Interstitial inflammation varied but was generally seen in areas of fibrosis. No evidence of immune complex deposition was detected using immunofluorescence of complement and immunoglobulin components. Electron microscopy, performed on 2 biopsies, showed evidence of endothelial cell injury. Significant glomerular basement membrane changes or glomerular inflammation were not detected.

### Abnormal Intracellular Localization of ALG5 in Affected Kidney Biopsy

**Figure 5:**
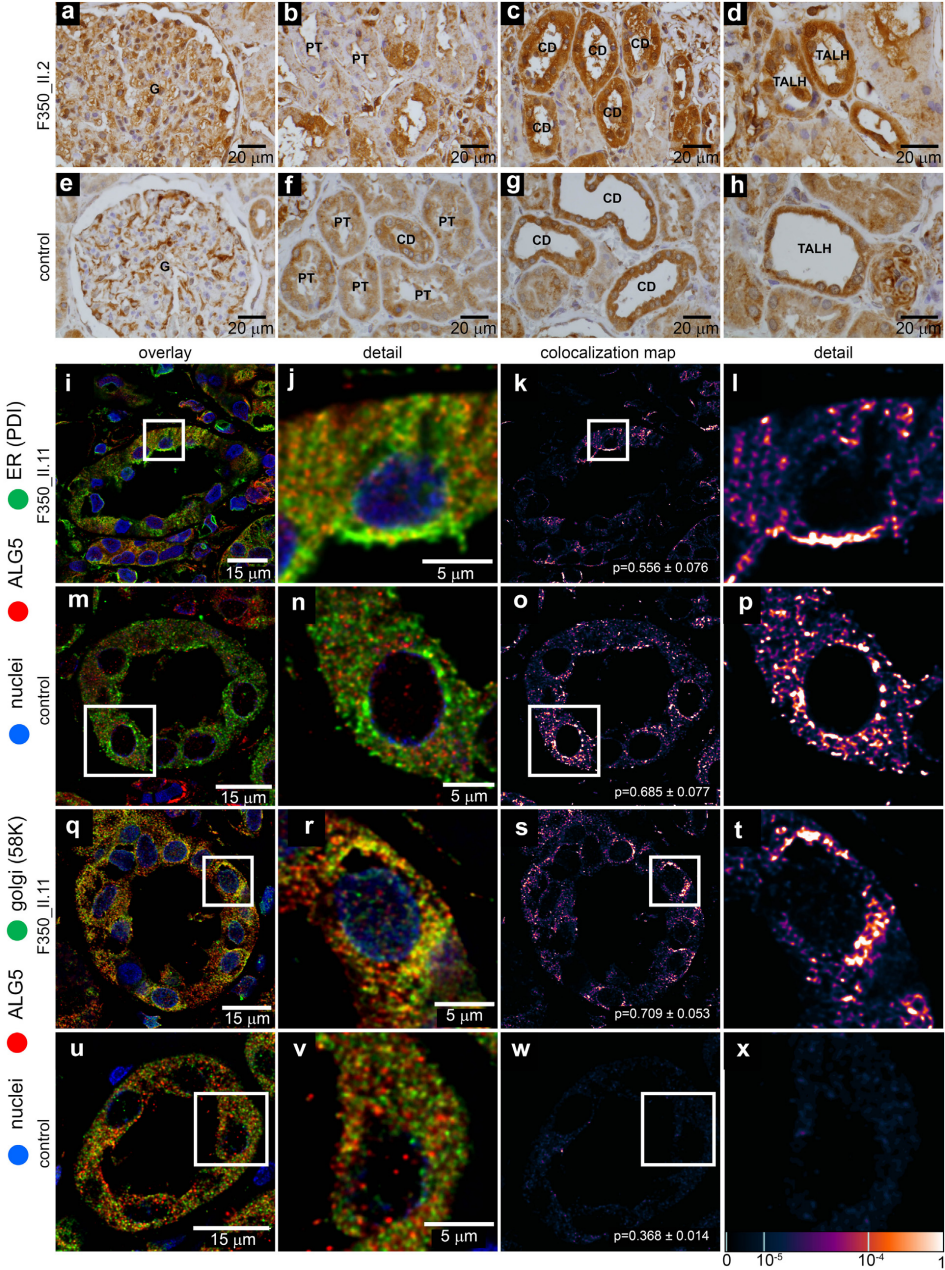
Detection of ALG5 in kidney biopsy. (a–d) ALG5 staining in genetically affected individual F350_II.2 with the missense variant p.R79W in ALG5 and (e–h) in a control subject. ALG5 protein was detected in all segments of nephron-glomerulus (G), proximal tubules (PT), distal tubules (DT), collecting ducts (CD), and thick ascending loop of Henle cells (TALH). In the affected subject was observed the coarsely granular intracytoplasmic staining of ALG5 compared to the finely granular and less intense staining in the control subject. (i–x) Intracellular localization of ALG5 in kidney biopsy. In affected kidney biopsy, parallel staining of ALG5 with Protein disulfide isomerase (PDI) (i in detail j), a marker of endoplasmic reticulum (ER), and with 58K Golgi-protein (q in detail r) demonstrate expected albeit less intense localization of the ALG5 in the ER (k in detail l) and pathologic localization in the Golgi (s in detail s). In the control kidney, parallel staining with PDI (m in detail n) and 58K Golgi protein (u in detail v) demonstrated localization of ALG5 in ER (o in detail p) but not in the Golgi (w in detail x). In the lower right corner of each image (5K, 5O, 5S, 5W) is Pearson coefficient ± SEM to show mean degree of colocalization between individual compartment markers and ALG5 in all analyzed tubules. The degree of ALG5 colocalization with selected markers is presented as the fluorescent signal overlap coefficient values that range from 0 to 1. The corresponding lookup table displays the resulting overlap coefficient values as the pseudo-color scale.

ALG5 immunostaining studies in 3 human kidney biopsies from genetically affected carriers from family F350 revealed granular positivity in all segments of nephron—glomeruli, proximal tubules, the thick ascending loop of Henle, and cortical collection ducts. In the affected subjects were observed the coarsely granular intracytoplasmic staining of ALG5 compared to the finely granular and less intense staining in the control subjects (Figure 5a–h). Parallel immunofluorescence staining of ALG5 and specific protein markers of secretory pathway compartments—ER, ER-Golgi intermediate compartment, Golgi apparatus, and plasma membrane showed abnormal distribution of ALG5 in Golgi apparatus and in the ER. In control kidney pathologic specimens, ALG5 was located exclusively in the ER (Figure 5i–x; Supplementary Figure S2A–P).

### ALG5 Variant Affects the Expression of N-Glycosylated Uromodulin but Not That of O-Glycosylated Mucin 1

**Figure 6:**
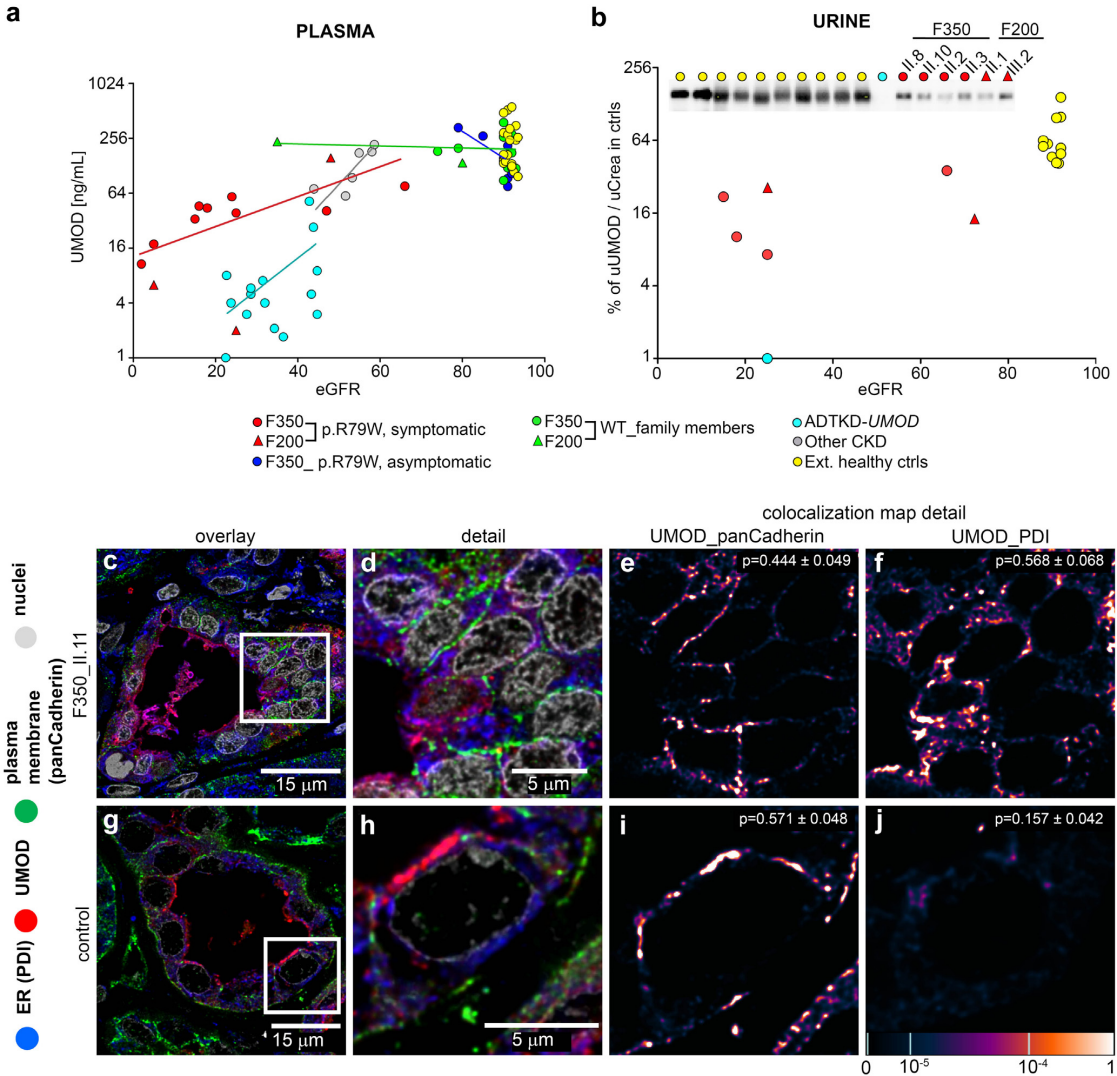
Uromodulin (UMOD) investigations. (a) Plasma concentration of UMOD decreases with reduced estimated glomerular filtration rate (ml/min per 1.73 m^2^) in individuals affected with the missense variant p.R79W in ALG5, individuals with UMOD pathogenic variants and individuals with unspecified CKD. The decrease in UMOD concentration is slower and less expressive in cases with the ALG5 variant than in cases with UMOD mutations. A best-fit line is included for each group (b) Semiquantitative and qualitative detection of urinary UMOD. Western blot of spot urine samples from 10 healthy controls, 1 patient with a canonical variant in *UMOD* (ADTKD-*UMOD*), and 6 genetically affected individuals from family F350 and family F200 showed a reduced amount of urinary UMOD in all investigated patients compared to healthy controls. No abnormality in electrophoretic mobility of residual UMOD suggestive of abnormal N-linked glycosylation was observed in patients. The sample volume was normalized to urinary creatinine level. Intracellular localization of UMOD in patients and control kidney biopsy. To elucidate the cause of the reduction of urinary excretion and the plasma level of UMOD, we analyzed its intracellular localization in kidney biopsy from the patient and control. In affected kidney biopsy, parallel staining of uromodulin (UMOD) with Protein disulfide isomerase (PDI), a marker of endoplasmic reticulum (ER), and with pan-Cadherin, (c in detail d a marker of the plasma membrane (PM)) demonstrate localization of the uromodulin in both, (d) partly in the ER (34%) and (f) partly on PM (66%). In the control kidney, (g in detail h) parallel staining of UMOD with PDI and pan-Cadherin demonstrated (i) localization of UMOD on PM (92%) (j) but not in the ER. In the upper right corner of each image (6K, 6F, 6I, 6J) is the Pearson coefficient ± SEM to show mean degree of colocalization between individual compartment markers and ALG5 in all analyzed tubules. The degree of UMOD colocalization with selected markers is demonstrated by the fluorescent signal overlap coefficient values ranging from 0 to 1. The resulting overlap coefficient values are presented as the pseudo-color, which scale is shown in the corresponding lookup table.

In addition to abnormal intracellular ALG5 deposition, we found pathological retention of N-glycosylated, and GPI modified uromodulin in the ER of kidney thick ascending loop of Henle cells of genetically affected individuals. In addition, plasma and urinary levels of uromodulin were reduced (Figure 6), whereas plasma levels and intracellular localization of O-glycosylated mucin 1 appeared to be normal (Supplementary Figure S3A–D).

### ALG5 Variant Does Not Affect Glycosylation of Transferrin

Transferrin is a clinically validated biomarker of congenital glycosylation disorders.^31^ However, profiling of this biomarker using Western blot did not reveal any abnormalities (Supplementary Figure S4).

### Proteomic and Glycoproteomic Profiles of Plasma

**Figure 7:**
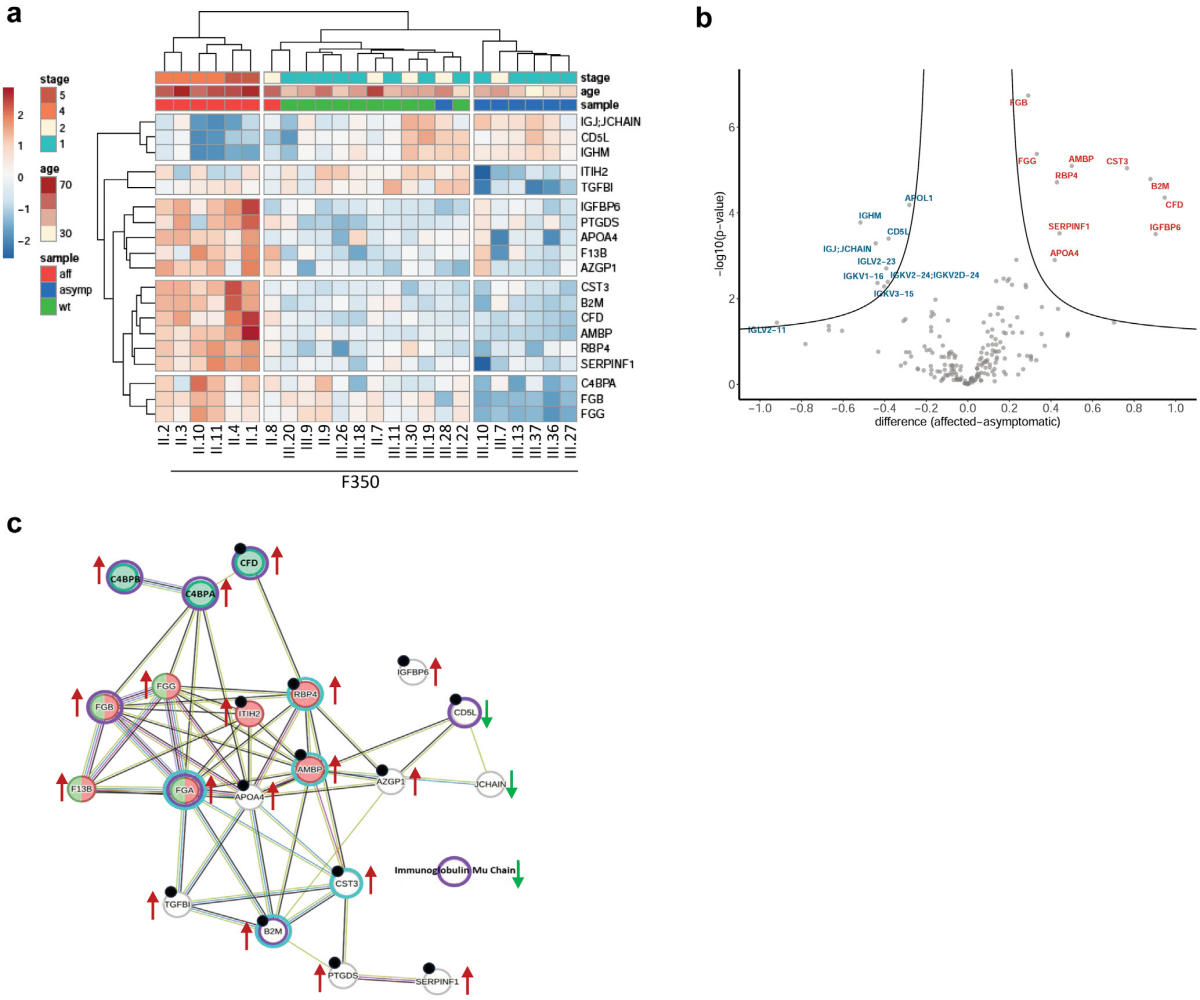
Proteomic analysis of plasma. (a) Unsupervised hierarchical clustering of 19 proteins identified by mass spectrometry and analysis of variance with significantly different abundance between samples of genetically affected individuals with CKD (aff), genetically affected individuals with normal kidney function CKD (asymp) and genetically unaffected family members (wt) from F350. Values are shown as Z-scores, and together with CKD stage and age of participants, are presented as the color codes, which scale is shown in the corresponding lookup tables. (b) Volcano plot demonstrating differences in plasma proteins abundancies identified by mass spectrometry between genetically affected individuals with CKD (aff) and genetically affected individuals with normal kidney function CKD (asymp). The X-axis shows the difference as a log_2_ value of a ratio of Z-scores of individual proteins; the Y-axis shows the statistical significance of the difference. (c) Physical interactions of proteins with significantly different abundance between genetically affected individuals with CKD and genetically affected individuals with normal kidney function CKD visualized by STRING database. Color circles depict proteins associated with immune response (Violet - DISGENET database), proteins associated with kidney disease, kidney insufficiency or kidney injury (Turquoise - DISGENET database), proteins associated with hemostasis (Red - STRING database), proteins associated with complement and coagulation cascade (Green - STRING database), proteins which were not associated with any of the listed category in STRING or DISGENET database (Gray) and proteins associated with either stage of CKD or progression of CKD or ESKD from the literature (Black dots). Arrows indicate an increase or decrease in corresponding protein abundance in genetically affected individuals with CKD. CKD, chronic kidney disease.

Proteomic profiling may reveal specific ALG5 dysfunction-related processes and factors associated with CKD progression. Therefore, plasma samples of 6 individuals with heterozygous R79W-ALG5 variant^*-*^ and CKD (clinically affected), 8 individuals with heterozygous R79W-ALG5 variant and an eGFR >60 ml/min per 1.73 m^2^ (clinically asymptomatic) and 10 family members with WT-ALG5 (genetically unaffected) from F350 were analyzed (Figure 7). Proteomic analysis identified 198 proteins (Supplementary Table S2). Unsupervised clustering analysis separated samples into 3 major clusters. The first cluster included all clinically affected individuals with heterozygous R79W-ALG5 variant. The second cluster included clinically unaffected individuals with heterozygous R79W-ALG5 variant. The third cluster included all genetically unaffected family members with WT-ALG5 and normal eGFR (Figure 7a). Twenty-one proteins differed significantly in abundance between clinically affected and clinically unaffected individuals with WT-ALG5 (Figure 7b and Supplementary Table S3).S1–S10 Functional analysis revealed these proteins are involved in the complement and coagulation cascade (CFD, C4BPA, C4BPB, FGA, FGB, FGG, F13B, and JCHAIN), inflammatory response (B2M, CD5L, CFD, C4BPA, C4BPB, FGA, and FGG), and hemostasis (AMBP, FGA, FGB, FGG, F13B, and RBP4,) and are associated with kidney diseases (AMBP, APOA4, AZGP1, B2M, CD5L, CFD, CST3, FGA, IGFBP6, ITIH2, PTGDS, RBP4, SERPINF1, and TGFBI) (Figure 7c). There was no statistically significant difference in individual protein abundance between family members with heterozygous R79W-ALG5 variant and normal eGFR and genetically unaffected family members (data not shown).

**Figure 8:**
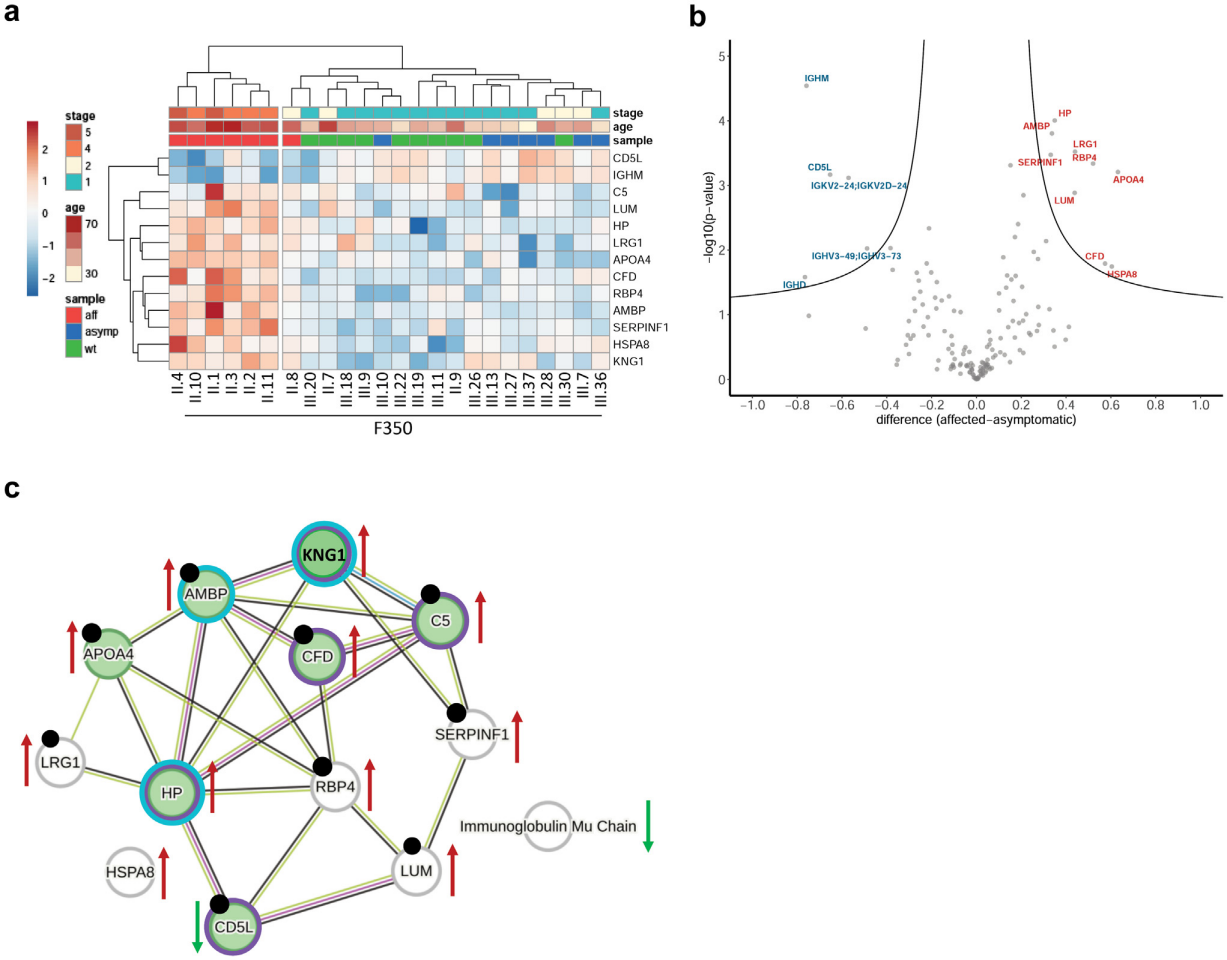
Glycoproteomic analysis of plasma. (a) Unsupervised hierarchical clustering of 12 glycoproteins identified by mass spectrometry and analysis of variance with significantly different abundance between samples of genetically affected individuals with CKD (aff), genetically affected individuals with normal kidney function CKD (asymp) and genetically unaffected family members (wt) from F350. Values are shown as Z-scores, and together with CKD stage and age of participants, are presented as the color codes, which scale is shown in the corresponding lookup tables. (b) Volcano plot demonstrating differences in plasma proteins abundancies identified by mass spectrometry between genetically affected individuals with CKD (aff) and genetically affected individuals with normal kidney function CKD (asymp). The X-axis shows the difference as a log_2_ value of a ratio of Z-scores of individual proteins; the Y-axis shows the statistical significance of the difference. (c) Physical interactions of proteins with significantly different abundance between genetically affected individuals with CKD and genetically affected individuals with normal kidney function CKD visualized by STRING database. Color circles depict proteins associated with immune response (Violet - DISGENET database), proteins associated with kidney disease, kidney insufficiency or kidney injury (Turquoise - DISGENET database), proteins associated with hemostasis (Red - STRING database), proteins associated with complement and coagulation cascade (Green – STRING database), proteins which were not associated with any of the listed category in STRING or DISGENET database (Gray) and proteins associated with either stage of CKD or progression of CKD or ESKD from the literature (Black dots). Arrows indicate an increase or decrease in corresponding protein abundance in genetically affected individuals with CKD. CKD, chronic kidney disease.

Individual with heterozygous R79W-ALG5 and CKD also differed from clinically unaffected and genetically unaffected individuals in glycoproteomic profiles (Figure 8a). Glycoproteomic analysis identified 154 proteins (Supplementary Table S4). Twelve glycoproteins differed significantly in abundance between clinically affected and clinically unaffected heterozygous R79W-ALG5 individuals (Figure 8b and Supplementary Table S5).S9–S16 All these proteins are involved in the complement and coagulation cascade (AMBP, APOA4, CD5L, CFD, HP, and KNG1), inflammatory response (CD5L, CFD, HP, and KNG1), protein folding (HSPA8) and are associated with kidney diseases (AMBP, APOA4, CD5L, CFD, HP, KNG1, LRG1, LUM, SERPINF1, and RBP4) (Figure 8c).

## Discussion

In this investigation, we report the clinical, structural, histopathologic, and functional correlates of a novel monoallelic missense variant in *ALG5* that we identified in 23 individuals from 2 multiplex Irish families sharing a related ancestor. Consistent with the one previous initial report of *ALG5* variants,^6^ affected individuals showed nonenlarged polycystic kidneys, tubulointerstitial atrophy and fibrosis, and slowly progressive CKD. Genetically affected individuals first developed an eGFR <60 ml/min per 1.73 m^2^ after age 50 years. At the most recent follow-up, 5 individuals had progressed to ESKD at an average age of 73 years. Compared with ADPKD, cystic changes did not appear severe enough to account for the degree of kidney failure. Moreover, 2 individuals (F200_II.5: eGFR 57 ml/min per 1.73 m^2^ [age 67 years] and F350_II.7: eGFR 79 ml/min per 1.73 m^2^ [age 69 years]) were found to have a few kidney and liver cysts, but they were not carrying the heterozygous R79W-ALG5 variant. Exome sequencing did not identify a pathogenic cystogenic variant that could account for their phenotype, and subsequent identity-by-descent estimates confirmed full ship relatedness (Supplementary Table S1). Although we cannot rule out the possibility that additional genetic and environmental modifiers contribute to a markedly milder phenotype,^32^ a similar observation was identified in a family with established ADPKD-*GANAB*,^33^ where it is possible that age-related increase in the simple kidney cysts, which are linked to impaired kidney function and CKD, may have contributed to cysts formation.^32^^,^^34^

**Figure 9:**
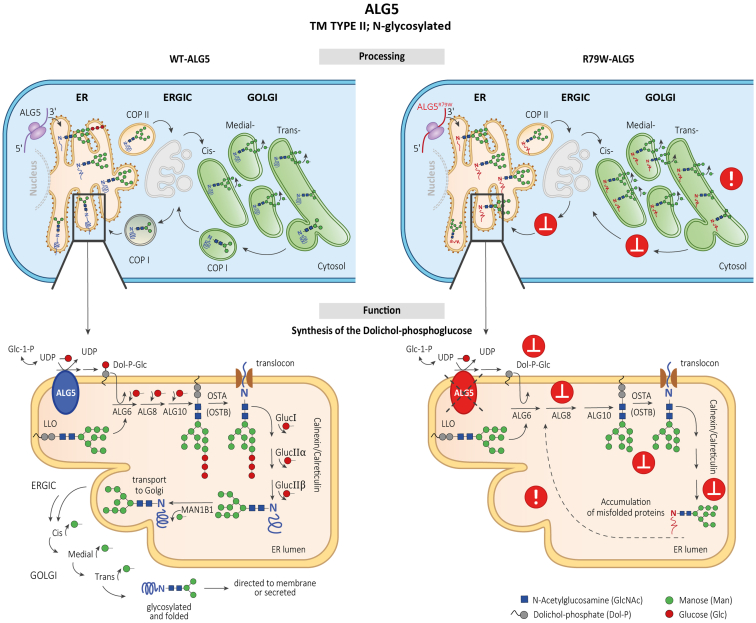
Hypothetical pathogenetic mechanisms of monoallelic *ALG5* variants. Upper left cartoon: ALG5 is an N-glycosylated protein that localizes in the endoplasmic reticulum (ER) membrane. Its biosynthesis proceeds through cotranslational translocation into ER and posttranslational modifications, including sequential maturation of N-linked oligosaccharides and folding in the ER, ERGIC, and Golgi apparatus. Fully maturated and properly folded ALG5 is finally retrieved back to the ER. Lower left cartoon: ALG5 catalyzes on the cytoplasmic face of ER membrane synthesis of dolichyl beta-D-glucosyl phosphate (Dol-P-Glc) from dolichyl phosphate and Uridine 5'-diphospho (UDP)-alpha-D-glucose. Dol-P-Glc is subsequently translocated by flippase into the ER lumen, where it provides glucose (Glc) residues for the sequential building of the growing lipid linked-oligosaccharides (LLO) by ALG6, ALG8, and ALG10. The resulting oligosaccharide glycan core consists typically of Man_9_Glc_3_GlcNAc_2_; The Man_9_Glc_3_GlcNAc_2_ oligosaccharide is transferred to the asparagine (N) residue of the polypeptide chain by an oligosaccharyltransferase complex OSTA that glycosylates nascent polypeptides traversing the translocon or OSTB that glycosylates proteins in the ER lumen. Then, folding of N-linked glycoproteins proceeds via subsequent removal of 3 Glc residues by glucosidases (Gluc I, Gluc IIα, and Gluc IIβ) and one mannose (Man) residue by ER mannosidase (MAN1B1) with assistance of chaperones (calnexin, calreticulin). Properly folded and Man_8_GlcNAc_2_ modified glycoproteins then traverse via COP II coated vesicles through ER-Golgi intermediate compartment (ERGIC) to the Golgi for final processing and sorting for transport to their eventual destinations. Upper right cartoon: the p.R79W variant affects the maturation, folding, and trafficking of the ALG5, leading to aberrant protein deposition within the Golgi apparatus and disturbance of Golgi homeostasis. Lower right cartoon: the p.R79W variant and ALG5 haploinsufficiency affects synthesis and structure of the LLO oligosaccharides with negative consequences on maturation, trafficking, and intracellular localization of other N-glycosylated proteins.

The *ALG5* variant identified in all clinically affected individuals has not been reported in the general population, affects an evolutionarily conserved residue, and is similar to other pathogenic missense *ALG5* variants identified in the 5 other reported families.^6^ The variant alters 1 of the arginine residues in the A-loop of the enzyme that is governing a dolichyl-phosphate beta-glucosyltransferase activity. In addition, we showed that the ALG5 protein is abnormally distributed in the Golgi apparatus of tubular cells in kidney biopsies from affected individuals. We also detected abnormal accumulation of uromodulin in the ER in kidney biopsies from affected individuals and decreased uromodulin urinary excretion and plasma concentration in affected individuals. We did not find evidence for a functional disorder of protein glycosylation, because the biosynthesis of transferrin, a clinically validated biomarker of congenital N-glycosylation disorders and of O-glycosylated mucin-1 were apparently normal. Figure 9 shows that ALG5 is a glycoprotein that is N-glycosylated on the asparagine residue 281. The protein is normally localized to the ER membrane and catalyzes synthesis on the cytosolic side of the ER of dolichol-phosphoglucose from dolichol phosphate and UDP-glucose.^28^ ER resident glycoproteins, such as ALG5, are synthesized in the ER and undergo posttranslational modifications, including N-linked glycosylation, chaperone-associated folding, and the maturation of N-linked oligosaccharides in the Golgi apparatus; and when fully maturated, are retrieved back into ER.^35^ Abnormal intracellular distribution of ALG5 in the affected kidney suggests that the p.R79W variant affects normal maturation and trafficking of the mutated protein. This pathogenetic mechanism may affect the retrograde recycling of ALG5, leading to aberrant protein deposition within the Golgi apparatus, disturbance of Golgi homeostasis, and Golgi stress-associated disease^36^ with consequences for maturation, trafficking, and intracellular localization of other N-glycosylated proteins.

Abnormal and impaired N-glycosylation of PC1 likely affects normal cell-cell interactions and induces cyst formation, as is seen in ADPKD-*PKD1* and *PKD2*. Although abnormal glycosylation leads to cystic changes, they are mild in comparison to those found in ADPKD and do not appear alone enough to lead to ESKD. However, defects in N-glycosylation of other proteins and impaired proteostasis in tubular cells could lead to accelerated apoptosis, tubular cell death, nephron dropout, and CKD.^37^ We showed that the ALG5 mutation affected uromodulin trafficking, with abnormal deposition of uromodulin within the ER. Abnormal deposition of uromodulin has been identified as a consistent pathologic finding in ADTKD-*UMOD*,^38^ where the clinical phenotype of tubulointerstitial fibrosis and CKD is similar to ADPKD-*ALG5*. Thus, we hypothesize a 2-fold pathophysiologic process, where aberrant glycosylation of PC1 and polycystin-2 leads to decreased PC1 and polycystin-2 surface expression, further leading to cystic disease. In addition, aberrant maturation and trafficking of PC1, polycystin-2, ALG5 itself, UMOD, and other N-glycosylated glycoproteins affect their intracellular distribution and lead to organelle stress. Identification and effects of the aberrant distribution of other proteins is important for therapeutic reasons. For example, if the effect of *ALG5* variant on uromodulin synthesis leads to CKD, the downregulation of uromodulin production could be a potential therapy for patients with ADPKD-*ALG5* (as well as ADPKD-*ALG8*).

ALG5 is involved in glycosylation of proteins throughout the body, and the restriction of clinical manifestations to the kidney may be related to the low turnover rate of tubular epithelial cells. These cells may be at increased risk of organelle stress over a much longer cell lifespan. Alternatively, aberrant distribution of specific proteins expressed only in the kidney (e.g., uromodulin) may, for some reason, be singularly pathogenic. Further work is needed in this area to explain the kidney-specific functional effects of variants in proteins synthesized throughout the body, including mucin-1 and ALG5.

In our investigation, we also sought to identify biomarkers for the biochemical diagnosis of ALG5 dysfunction and kidney disease progression. We found normal electrophoretic profiles of plasma transferrin in genetically affected individuals, indicating that residual ALG5 activity does not impede the biosynthesis of this validated clinical biomarker of congenital N-glycosylation disorders. However, it does not rule out the prospect that the composition of their N-linked oligosaccharide chains may be changed.

Proteomics and glycoproteomic profiling were not informative in identifying specific biomarkers of ALG5 dysfunction, because there was no significant difference in individual proteins between genetically affected individuals with normal eGFR and genetically unaffected family members. Genetically affected individuals with reduced eGFR demonstrated well-documented upregulation of kidney insufficiency-associated proteins, but no ALG5-specific biomarker was identified.

Our findings confirm previous studies where dysfunction of ALG5^6^ and other proteins involved in glycoprotein biosynthesis^33^^,^^39^^,^^40^ have a dominant negative effect on organelle functionality, the maturation, trafficking of N-glycosylated and/or GPI-anchored proteins, and kidney function.

### Conclusions

We report a novel pathogenic *ALG5* variant causing atypical cystic kidneys, tubulointerstitial fibrosis, and late-onset ESKD, which validates and complements the findings from the initial study identifying pathogenic *ALG5* variants.^6^ Our findings provide new insights into the pathogenesis of this condition: monoallelic pathogenic *ALG5* variants affect maturation, trafficking, and intracellular localization of ALG5 and another N-glycosylated and GPI-anchored protein uromodulin, leading to structural and functional changes in the kidney.

## Disclosure

EE is sponsored with StAR PhD fellowship RCSI -Ireland. HaH is funded by ERA Net for Research on Rare Diseases and Cooperatio-Pediatrics funding. VS is funded by the grant: the application of new methods of genomic analysis in cases of rare genetic based diseases with negative results of genetic and genomic analyses. Changes of transcriptome during early postnatal development in humans: impact of premature birth on control of energy metabolism, both provided by Ministry of Health, Czech Republic. GLC has research funding from Biomarin. PJC is a member of Irish Health Research Board. All the other authors have declared no competing interests.

## Supplementary Material

Supplementary File (PDF) **Supplementary Methods. Supplemental References. Figure S1.** Overall kidney survival and kidney / liver cystic phenotypes of patients with heterozygous R79W-ALG5 variant. **Figure S2.** Intracellular localization to endoplasmic reticulum–Golgi intermediate compartment (ERGIC) and plasma membrane (PM) of ALG5 in kidney biopsy. **Figure S3.** Plasma mucin-1 (CA15-3) levels in family members with heterozygous R79W-ALG5 variant and WT-ALG5 and intracellular localization of MUC1 in patients’ and control’s kidney biopsies. **Figure S4.** Western bot of plasma transferrin of affected individuals with heterozygous R79W-ALG5 variant from F350 in comparison to individual with glycosylation disorder (mutation in *PMM2*, CDG type 1a). **Table S1.** PLINK identity-by-descent (IBD) analysis. **Table S2.** Proteomic analysis identified 198 proteins. **Table S3.** Proteomic analysis identified 19 dysregulated proteins in genetically affected individuals with advanced CKD (stage 4 and 5) compared to genetically affected individuals with early CKD (stage 1–3) and genetically unaffected family members. **Table S4.** Glycoproteomic analysis identified 154 proteins. **Table S5.** Glycoproteomic analysis identified 12 dysregulated proteins in genetically affected individuals with advanced CKD (stage 4 and 5) compared to genetically affected individuals with early CKD (stage 1–3) and genetically unaffected family members. **STROBE Checklist.**

## Footnotes

Supplementary File (PDF)

**Supplementary Methods.**

**Supplemental References.**
**Figure S1.** Overall kidney survival and kidney / liver cystic phenotypes of patients with heterozygous R79W-ALG5 variant. **Figure S2.** Intracellular localization to endoplasmic reticulum–Golgi intermediate compartment (ERGIC) and plasma membrane (PM) of ALG5 in kidney biopsy. **Figure S3.** Plasma mucin-1 (CA15-3) levels in family members with heterozygous R79W-ALG5 variant and WT-ALG5 and intracellular localization of MUC1 in patients’ and control’s kidney biopsies. **Figure S4.** Western bot of plasma transferrin of affected individuals with heterozygous R79W-ALG5 variant from F350 in comparison to individual with glycosylation disorder (mutation in *PMM2*, CDG type 1a). **Table S1.** PLINK identity-by-descent (IBD) analysis. **Table S2.** Proteomic analysis identified 198 proteins. **Table S3.** Proteomic analysis identified 19 dysregulated proteins in genetically affected individuals with advanced CKD (stage 4 and 5) compared to genetically affected individuals with early CKD (stage 1–3) and genetically unaffected family members. **Table S4.** Glycoproteomic analysis identified 154 proteins. **Table S5.** Glycoproteomic analysis identified 12 dysregulated proteins in genetically affected individuals with advanced CKD (stage 4 and 5) compared to genetically affected individuals with early CKD (stage 1–3) and genetically unaffected family members.
**STROBE Checklist.**
