# Supplementary material for: A Novel Monoallelic ALG5 Variant Causing Late-Onset ADPKD and Tubulointerstitial Fibrosis
Source: Kidney Int Rep. 2024 Apr 15;9(7):2209–26. doi: 10.1016/j.ekir.2024.04.031 (PMC11284371; doi:10.1016/j.ekir.2024.04.031)
Supplement: Supplementary File (PDF) — Supplementary Methods. Supplemental References. Figure S1. Overall kidney survival and kidney / liver cystic phenotypes of patients with heterozygous R79W-ALG5 variant. Figure S2. Intracellular localization to endoplasmic reticulum–Golgi intermediate compartment (ERGIC) and plasma membrane (PM) of ALG5 in kidney biopsy. Figure S3. Plasma mucin-1 (CA15-3) levels in family members with heterozygous R79W-ALG5 variant and WT-ALG5 and intracellular localization of MUC1 in patients’ and control’s kidney biopsies. Figure S4. Western bot of plasma transferrin of affected individuals with heterozygous R79W-ALG5 variant from F350 in comparison to individual with glycosylation disorder (mutation in PMM2, CDG type 1a). Table S1. PLINK identity-by-descent (IBD) analysis. Table S2. Proteomic analysis identified 198 proteins. Table S3. Proteomic analysis identified 19 dysregulated proteins in genetically affected individuals with advanced CKD (stage 4 and 5) compared to genetically affected individuals with early CKD (stage 1–3) and genetically unaffected family members. Table S4. Glycoproteomic analysis identified 154 proteins. Table S5. Glycoproteomic analysis identified 12 dysregulated proteins in genetically affected individuals with advanced CKD (stage 4 and 5) compared to genetically affected individuals with early CKD (stage 1–3) and genetically unaffected family members. STROBE Checklist. [file mmc1.pdf]

## Supplementary Material

This appendix has been provided by the authors to give readers additional information about their work.

**Supplement to: Elhussein A. E. Elhassan, Tereza Kmochová et al. A Novel Monoallelic *ALG5* Variant Causing Late-onset ADPKD and Tubulointerstitial Fibrosis**

**Table of contents**

**Figure S1** *(p. 3)*
Overall kidney survival and kidney / liver cystic phenotypes of patients with heterozygous R79W-ALG5 variant

**Figure S2** *(p. 4)*
Intracellular localization to endoplasmic reticulum–Golgi intermediate compartment (ERGIC) and plasma membrane (PM) of ALG5 in kidney biopsy

**Figure S3** *(p. 5)*
Plasma mucin-1 (CA15-3) levels in family members with heterozygous R79W-ALG5 variant and WT-ALG5 and intracellular localization of MUC1 in patients' and control's kidney biopsies

**Figure S4** *(p. 6)*
Western bot of plasma transferrin of affected individuals with heterozygous R79W-ALG5 variant from F350 in comparison to individual with glycosylation disorder (mutation in *PMM2*, CDG type 1a)

**Table S1** *(p. 7)*
PLINK identity-by-descent (IBD) analysis

**Table S2** (*p. 8 – 15)*
Proteomic analysis identified 198 proteins

**Table S3** (*p. 16 – 17)*
Proteomic analysis identified 19 dysregulated proteins in genetically affected individuals with advanced CKD (stage 4 and 5) compared to genetically affected individuals with early CKD (stage 1 – 3) and genetically unaffected family members

**Table S4** (*p. 18 – 22)*
Glycoproteomic analysis identified 154 proteins

**Table S5** (p. *23)*
Glycoproteomic analysis identified 12 dysregulated proteins in genetically affected individuals with advanced CKD (stage 4 and 5) compared to genetically affected individuals with early CKD (stage 1 – 3) and genetically unaffected family members

**Supplementary methods** *(p. 24 - 28)*

**STROBE checklist** *(p. 29 – 32)*

**Supplemental references** *(p. 33 - 35)*

**Figure S1: Overall kidney survival and kidney / liver cystic phenotypes of patients with heterozygous R79W-ALG5 variant**

**A**

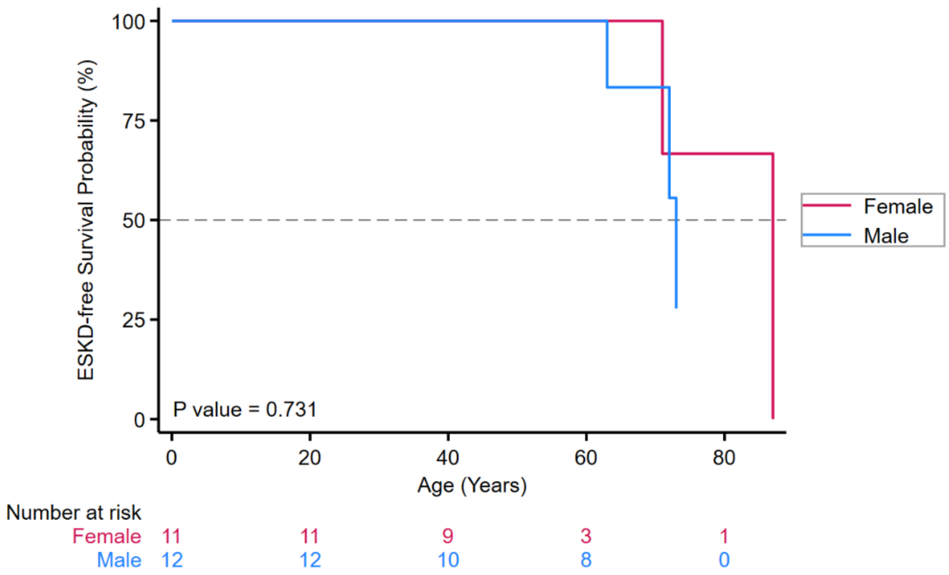


**B**

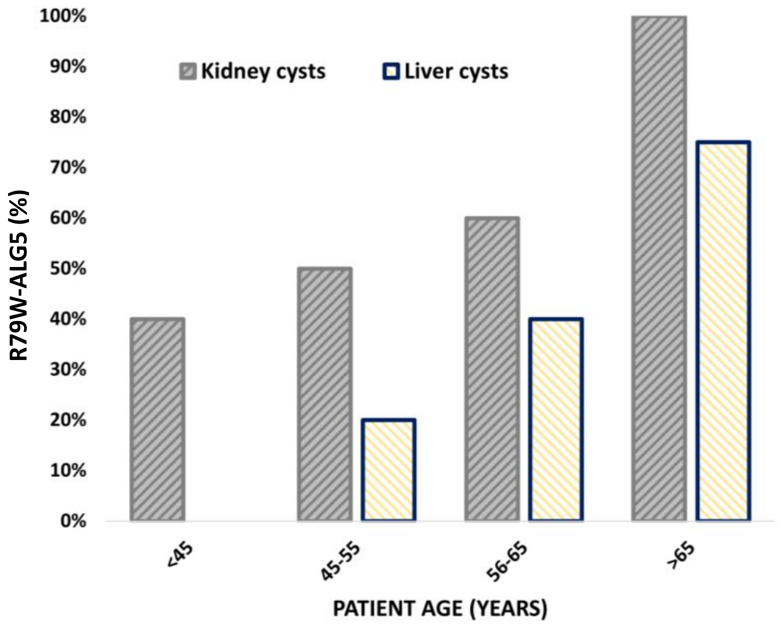


**A.** Probability of reaching kidney failure with replacement therapy in patients with disease-causing variant in *ALG5* demonstrates that renal survival does not differ between male and female subjects. **B.** Patients with the heterozygous R79W-ALG5 variant (R79W-ALG5) had a higher incidence of kidney and liver cysts as they age.

**Figure S2: Intracellular localization to endoplasmic reticulum–Golgi intermediate compartment (ERGIC) and plasma membrane (PM) of ALG5 in kidney biopsy**

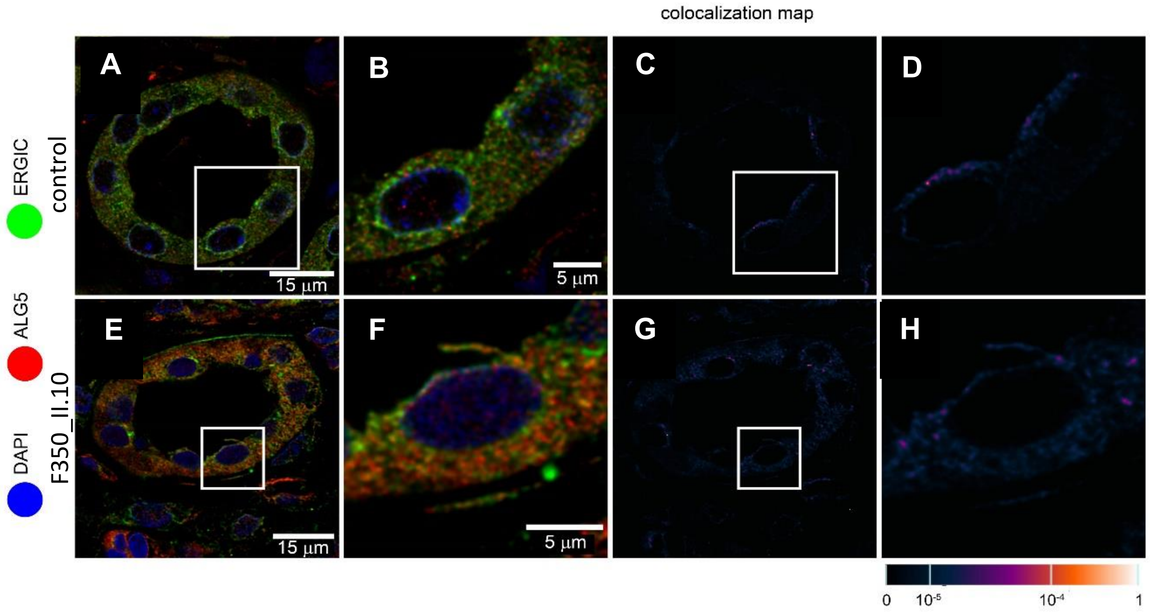


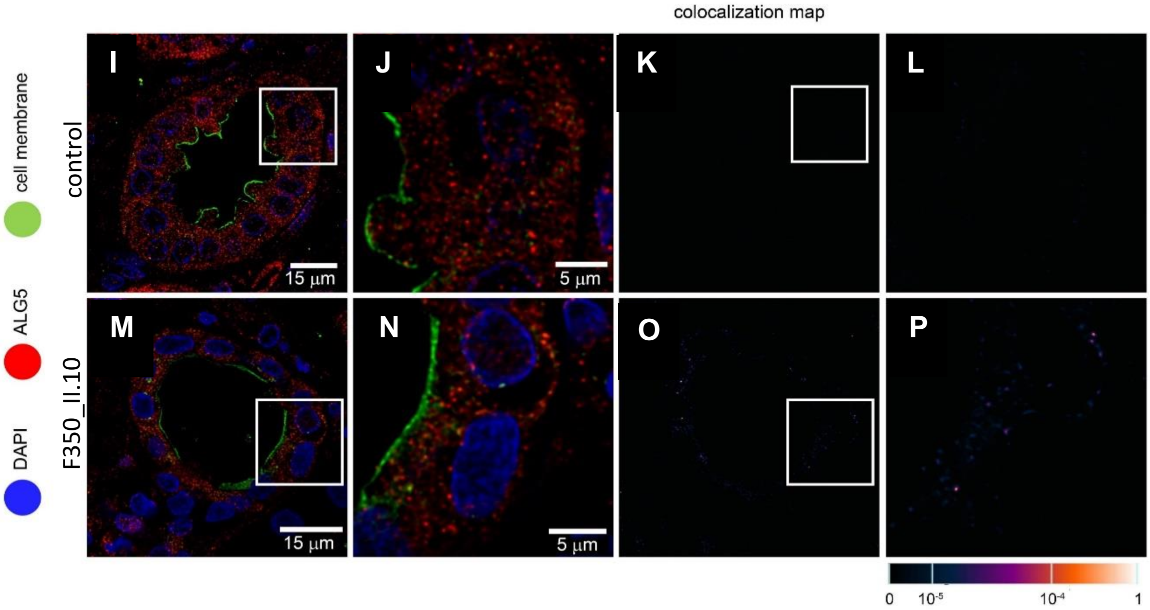


Parallel staining of ALG5 with monoclonal mouse anti-LMAN1 antibody, a marker of ERGIC **(A-H)**, and with pan-Cadherin, a marker of PM **(I-P)**, demonstrating the absence of mutant proteins in the ERGIC **(G in detail H)** as well as in the PM in kidney biopsy of affected individual **(K in detail L)** and control **(O in detail P)**. The degree of ALG5 colocalization with selected markers is presented as the fluorescent signal overlap coefficient values ranging from 0 to 1. The corresponding lookup table displays the resulting overlap coefficient values as the pseudo-color scale.

**Figure S3: Plasma mucin-1 (CA15-3) levels in family members with heterozygous R79W-ALG5 and WT-ALG5 and WT-ALG5 and intracellular localization of MUC1 in patients' and control's kidney biopsies**

**S3A**

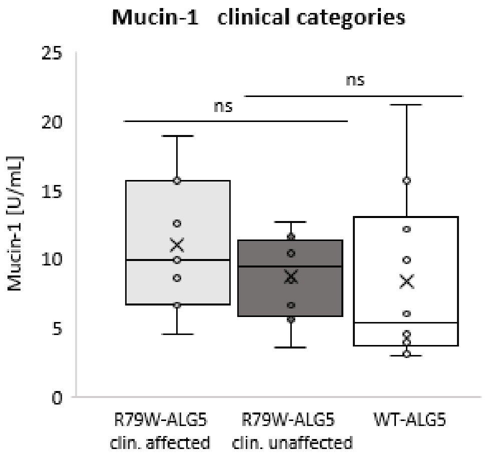


**S3B**

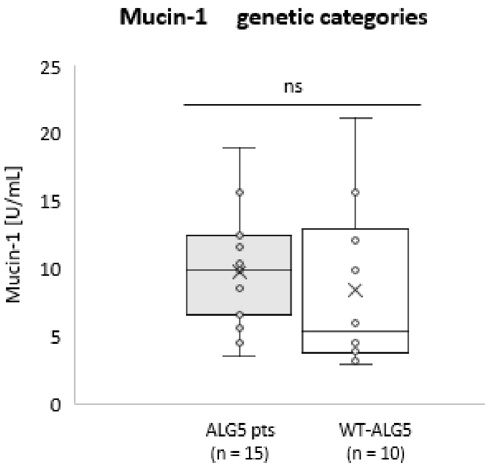


**S3C**

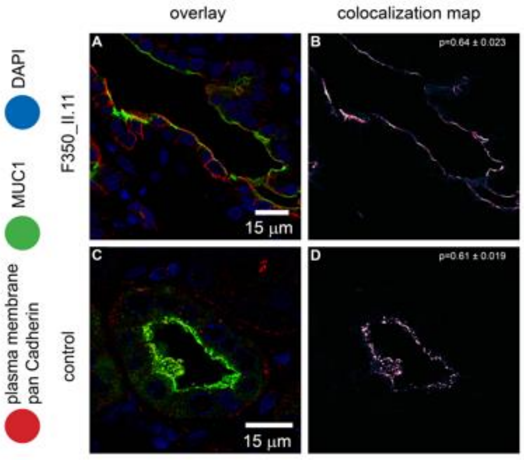


**S3D**

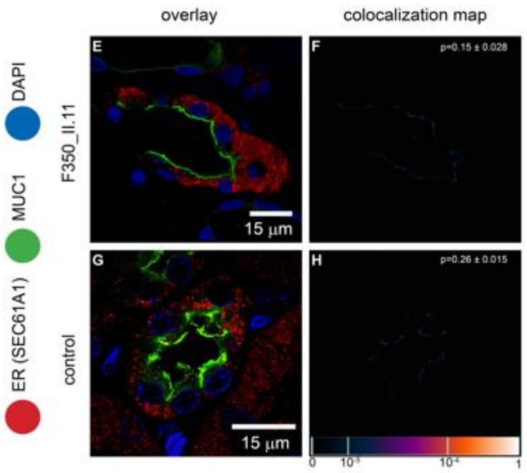


There is no difference in the mean of plasma mucin 1 (CA15-3) concentration between clinically affected, clinically unaffected family members with heterozygous R79W-ALG5 variant (n=10) and WT-ALG5 (student's t-test, $P \geq 0.05$) (**S3A**). In addition, there is no difference in mucin-1 levels between genetically affected family members with ALG5$^{R79W/-}$ (clinically affected and clinically unaffected as defined by the eGFR cutoff of 90 ml/min) and genetically unaffected family members (**S3B**). Also, intracellular localization of mucin-1 is not different in patients' and control's kidney specimen. In both is mucin-1 localized on the apical pole of plasma membrane of tubular cells (**S3C, S3D**).

**Figure S4: Western blot of plasma transferrin of affected individuals with heterozygous R79W-ALG5 from F350 in comparison to individual with glycosylation disorder (mutation in *PMM2*, CDG type 1a)**

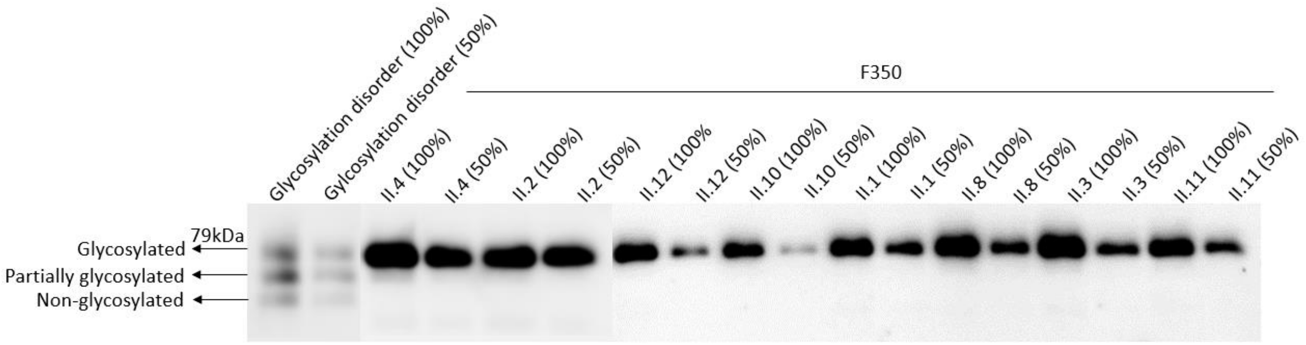


Western blot of plasma transferrin showed in affected individuals with heterozygous R79W-ALG5 variant$^{/-}$ one band corresponding to fully processed glycosylated transferrin (79kDa) while in individual with glycosylation disorder (mutation in *PMM2*, CDG type 1a) were detected three bands corresponding to non-glycosylated (74kDa), partially glycosylated (77kDa) and fully glycosylated (79kDa) forms of transferrin. The samples were loaded in two volumes normalized to total protein concentration (100% corresponds to 30 ng and 50% corresponds to 15 ng of total protein).

**Table S1: PLINK identity-by-descent (IBD) analysis. The IBD-sharing coefficients indicated that families F200 and F350 are related to one another.**

| ID 1 | ID 2 | Z0 | Z1 | Z2 | PI_HAT | Inferred Relationship Degree |
|---|---|---|---|---|---|---|
| F200_II.9 | F200_II.1 | 0.2336 | 0.5625 | 0.2039 | 0.4852 | First Degree (Full Sibling) |
| F350_II.6 | F200_II.1 | 0.5949 | 0.4051 | 0 | 0.2025 | Second Degree (eg. Half Sibling) |
| F350_II.6 | F200_II.9 | 0.6808 | 0.2918 | 0.0273 | 0.1733 | Third Degree (eg. First Cousin) |

The table indicates the PI_HAT score (a measure of overall IBD) and Z0, Z1, Z2 (the probability of having IBD of 0,1 or 2 respectively) between pairs of individuals. Parent-offspring relationships have (Z0, Z1, Z2 of 0,1,0) indicating that the individuals share approximately 50% IBD across the loci tested. Some degree of variance from the expected values for inferred relationships is common due to recombination events and technical errors. IBD-based coefficients confirmed a sibling-level (first-degree) relatedness between known siblings F200:II.9 and F200:II.1. Pairwise IBD estimates inferred second-degree and third-degree relationship estimates between F350_II.6 / F200_II.1 and F350_II.6 / F200_II.9, respectively. ID: identifier

**Table S2**

**Proteomic analysis identified 198 proteins**

| Protein name | Protein ID | Mol. weight [kDa] | Gene name | Score[a] | Intensities[b] |
|---|---|---|---|---|---|
| Actin, cytoplasmic 2 | P63261 | 41,792 | ACTG1 | 323,31 | 32 866 000 |
| Afamin | P43652 | 69,068 | AFM | 323,31 | 158 520 000 |
| Alpha-1-acid glycoprotein 1 | P02763 | 23,539 | ORM1 | 323,31 | 420 380 000 |
| Alpha-1-acid glycoprotein 2 | P19652 | 23,602 | ORM2 | 323,31 | 212 520 000 |
| Alpha-1-antichymotrypsin | P01011 | 47,65 | SERPINA3 | 323,31 | 181 060 000 |
| Alpha-1-antitrypsin | P01009 | 46,736 | SERPINA1 | 323,31 | 2 228 800 000 |
| Alpha-1B-glycoprotein | P04217 | 54,253 | A1BG | 323,31 | 387 710 000 |
| Alpha-2-antiplasmin | P08697 | 54,565 | SERPINF2 | 323,31 | 95 957 000 |
| Alpha-2-HS-glycoprotein | P02765 | 39,34 | AHSG | 323,31 | 683 860 000 |
| Alpha-2-macroglobulin | P01023 | 163,29 | A2M | 323,31 | 1 898 800 000 |
| Angiotensinogen | P01019 | 52,069 | AGT | 323,31 | 70 266 000 |
| Antithrombin-III | P01008 | 52,602 | SERPINC1 | 323,31 | 383 510 000 |
| Apolipoprotein A-I | P02647 | 30,777 | APOA1 | 323,31 | 4 493 400 000 |
| Apolipoprotein A-II | P02652 | 11,175 | APOA2 | 323,31 | 1 706 600 000 |
| Apolipoprotein A-IV | P06727 | 45,371 | APOA4 | 323,31 | 640 960 000 |
| Apolipoprotein B-100 | P04114 | 515,6 | APOB | 323,31 | 992 950 000 |
| Apolipoprotein C-I | P02654 | 8,647 | APOC1 | 151,89 | 60 661 000 |
| Apolipoprotein C-II | P02655 | 20,049 | APOC2 | 323,31 | 47 292 000 |
| Apolipoprotein C-III | P02656 | 10,852 | APOC3 | 323,31 | 198 150 000 |
| Apolipoprotein C-IV | P55056 | 14,553 | APOC4 | 5,7275 | 743 950 |
| Apolipoprotein D | P05090 | 21,275 | APOD | 323,31 | 70 491 000 |
| Apolipoprotein E | P02649 | 36,154 | APOE | 323,31 | 111 910 000 |
| Apolipoprotein F | Q13790 | 35,399 | APOF | 136,54 | 4 270 200 |
| Apolipoprotein L1 | O14791 | 43,974 | APOL1 | 323,31 | 48 718 000 |
| Apolipoprotein M | O95445 | 21,253 | APOM | 18,905 | 6 933 800 |
| Apolipoprotein(a) | P08519 | 226,54 | LPA | 323,31 | 89 154 000 |
| Attractin | O75882 | 158,54 | ATRN | 68,359 | 6 912 100 |
| Beta-2-glycoprotein 1 | P02749 | 38,298 | APOH | 323,31 | 539 430 000 |
| Beta-2-microglobulin | P61769 | 13,714 | B2M | 122,39 | 13 618 000 |
| Beta-Ala-His dipeptidase | Q96KN2 | 56,691 | CNDP1 | 7,4043 | 1 972 300 |
| Biotinidase | P43251 | 61,132 | BTD | 37,066 | 6 195 200 |
| C4b-binding protein alpha chain | P04003 | 67,033 | C4BPA | 323,31 | 160 270 000 |

| | | | | | |
|---|---|---|---|---|---|
| C4b-binding protein beta chain | P20851 | 28,357 | C4BPB | 112,02 | 22 227 000 |
| Carbonic anhydrase 1 | P00915 | 28,87 | CA1 | 65,419 | 2 896 700 |
| Carboxypeptidase B2 | Q96IY4 | 48,424 | CPB2 | 10,583 | 3 091 000 |
| Carboxypeptidase N catalytic chain | P15169 | 52,286 | CPN1 | 24,189 | 3 659 600 |
| Carboxypeptidase N subunit 2 | P22792 | 60,556 | CPN2 | 199,58 | 17 415 000 |
| CD44 antigen | P16070 | 81,537 | CD44 | 7,2265 | 583 620 |
| CD5 antigen-like | O43866 | 38,087 | CD5L | 323,31 | 47 801 000 |
| Ceruloplasmin | P00450 | 122,2 | CP | 323,31 | 923 730 000 |
| Clusterin | P10909 | 52,494 | CLU | 323,31 | 188 810 000 |
| Coagulation factor IX | P00740 | 51,778 | F9 | 9,9456 | 1 802 600 |
| Coagulation factor V | P12259 | 251,7 | F5 | 22,389 | 19 738 000 |
| Coagulation factor XI | P03951 | 70,108 | F11 | 16,516 | 776 600 |
| Coagulation factor XII | P00748 | 67,791 | F12 | 323,31 | 45 503 000 |
| Coagulation factor XIII A chain | P00488 | 83,267 | F13A1 | 19,299 | 3 616 800 |
| Coagulation factor XIII B chain | P05160 | 75,51 | F13B | 46,053 | 5 898 300 |
| Complement C1q subcomponent subunit A | P02745 | 26,016 | C1QA | 89,119 | 12 060 000 |
| Complement C1q subcomponent subunit B | P02746 | 26,721 | C1QB | 75,836 | 18 982 000 |
| Complement C1q subcomponent subunit C | P02747 | 25,773 | C1QC | 28,058 | 6 750 000 |
| Complement C1r subcomponent | P00736 | 80,118 | C1R | 323,31 | 84 963 000 |
| Complement C1r subcomponent-like protein | Q9NZP8 | 53,498 | C1RL | 8,9266 | 6 203 100 |
| Complement C1s subcomponent | P09871 | 76,684 | C1S | 323,31 | 52 473 000 |
| Complement C2 | P06681 | 83,267 | C2 | 48,532 | 11 010 000 |
| Complement C3 | P01024 | 187,15 | C3 | 323,31 | 2 503 100 000 |
| Complement C4-A | A0A0G2JPR0 | 192,87 | C4A | 323,31 | 1 272 500 000 |
| Complement C4-B | P0C0L5 | 192,75 | C4B | 192,42 | 28 320 000 |
| Complement C5 | P01031 | 188,3 | C5 | 323,31 | 38 499 000 |
| Complement component C6 | P13671 | 104,79 | C6 | 286,45 | 45 089 000 |
| Complement component C7 | P10643 | 93,517 | C7 | 323,31 | 19 806 000 |

| | | | | | |
|---|---|---|---|---|---|
| Complement component C8 alpha chain | P07357 | 65,163 | C8A | 323,31 | 41 920 000 |
| Complement component C8 beta chain | P07358 | 67,046 | C8B | 150,03 | 10 934 000 |
| Complement component C8 gamma chain | P07360 | 22,277 | C8G | 143,33 | 28 594 000 |
| Complement component C9 | P02748 | 63,173 | C9 | 323,31 | 49 055 000 |
| Complement factor B | P00751 | 140,94 | CFB | 323,31 | 457 420 000 |
| Complement factor D | P00746 | 27,033 | CFD | 26,772 | 4 116 900 |
| Complement factor H | A0A0D9SG88 | 51,007 | CFH | 150,23 | 3 316 400 |
| Complement factor H | P08603 | 139,09 | CFH | 323,31 | 632 150 000 |
| Complement factor H-related protein 1 | Q03591 | 37,65 | CFHR1 | 323,31 | 10 504 000 |
| Complement factor H-related protein 2 | P36980 | 39,415 | CFHR2 | 3,7337 | 1 655 300 |
| Complement factor I | P05156 | 65,75 | CFI | 323,31 | 41 049 000 |
| Corticosteroid-binding globulin | P08185 | 45,14 | SERPINA6 | 185,39 | 10 126 000 |
| Cystatin-C | P01034 | 15,799 | CST3 | 21,209 | 3 517 100 |
| Dynein heavy chain 3, axonemal | Q8TD57 | 470,77 | DNAH3 | 2,2531 | 31 187 000 |
| Extracellular matrix protein 1 | Q16610 | 60,673 | ECM1 | 50,871 | 7 366 700 |
| Fetuin-B | Q9UGM5 | 42,054 | FETUB | 63,286 | 1 365 300 |
| Fibrinogen alpha chain | P02671 | 94,972 | FGA | 323,31 | 3 395 800 000 |
| Fibrinogen beta chain | P02675 | 55,928 | FGB | 323,31 | 2 458 200 000 |
| Fibrinogen gamma chain | P02679 | 51,511 | FGG | 323,31 | 853 150 000 |
| Fibronectin | P02751 | 272,32 | FN1 | 323,31 | 237 880 000 |
| Fibulin-1 | B1AHL2 | 78,329 | FBLN1 | 63,915 | 5 611 000 |
| Fibulin-1 | P23142 | 77,213 | FBLN1 | 268,82 | 11 386 000 |
| Ficolin-3 | O75636 | 32,903 | FCN3 | 196,54 | 6 948 000 |
| Filamin-A | P21333 | 280,74 | FLNA | 160,27 | 8 689 500 |
| Galectin-3-binding protein | Q08380 | 65,33 | LGALS3BP | 178,62 | 10 068 000 |
| Gelsolin | P06396 | 85,696 | GSN | 323,31 | 192 510 000 |
| Gene | P01599 | 12,778 | | 4,5245 | 1 102 100 |
| Glutathione peroxidase 3 | P22352 | 25,552 | GPX3 | 5,9778 | 1 671 100 |

| | | | | | |
|---|---|---|---|---|---|
| Haptoglobin | P00738 | 45,205 | HP | 323,31 | 2 969 200 000 |
| Haptoglobin-related protein | P00739 | 39,029 | HPR | 194,65 | 33 971 000 |
| Hemoglobin subunit alpha | P69905 | 15,257 | HBA1 | 323,31 | 214 190 000 |
| Hemoglobin subunit beta | P68871 | 15,998 | HBB | 323,31 | 92 917 000 |
| Hemopexin | P02790 | 51,676 | HPX | 323,31 | 650 410 000 |
| Heparin cofactor 2 | P05546 | 57,07 | SERPIND1 | 118,33 | 12 620 000 |
| Hepatocyte growth factor activator | Q04756 | 70,681 | HGFAC | 25,069 | 3 523 300 |
| Hepatocyte growth factor-like protein | P26927 | 80,319 | MST1 | 13,288 | 2 120 400 |
| Histidine-rich glycoprotein | P04196 | 59,578 | HRG | 323,31 | 373 950 000 |
| Hyaluronan-binding protein 2 | Q14520 | 62,671 | HABP2 | 62,24 | 9 322 000 |
| Cholinesterase;Carboxylic ester hydrolase | P06276 | 68,417 | BCHE | 35,131 | 4 210 800 |
| Ig alpha-1 chain C region | P01876 | 37,654 | IGHA1 | 323,31 | 2 825 600 000 |
| Ig alpha-2 chain C region | P01877 | 36,591 | IGHA2 | 2,825 | 3 425 100 |
| Ig delta chain C region | P01880 | 42,352 | IGHD | 323,31 | 23 121 000 |
| Ig gamma-1 chain C region | P01857 | 36,105 | IGHG1 | 323,31 | 3 034 800 000 |
| Ig gamma-2 chain C region | P01859 | 35,9 | IGHG2 | 323,31 | 958 390 000 |
| Ig gamma-3 chain C region | P01860 | 41,287 | IGHG3 | 323,31 | 584 080 000 |
| Ig gamma-4 chain C region | P01861 | 35,94 | IGHG4 | 323,31 | 286 030 000 |
| Ig heavy chain V-III region DOB | P01782 | 12,945 | IGHV3-9 | 67,909 | 12 122 000 |
| Ig heavy chain V-III region JON | P01780 | 12,943 | IGHV3-7 | 61,72 | 15 316 000 |
| Ig kappa chain C region | P01834 | 11,765 | IGKC | 323,31 | 3 435 100 000 |
| Ig kappa chain V-I region BAN | P04430 | 12,618 | IGKV1-16 | 76,711 | 1 603 600 |
| Ig kappa chain V-II region FR | P01615 | 12,957 | IGKV2D-28 | 85,602 | 12 237 000 |
| Ig kappa chain V-II region RPMI 6410 | P06310 | 13,185 | IGKV2-30 | 5,2484 | 1 681 800 |
| Ig kappa chain V-III region B6 | P01619 | 12,557 | IGKV3-20 | 323,31 | 11 485 000 |

| | | | | | |
|---|---|---|---|---|---|
| Ig kappa chain V-III region POM | P01624 | 12,496 | IGKV3-15 | 25,113 | 13 921 000 |
| Ig kappa chain V-III region VG | P04433 | 12,575 | IGKV3D-11 | 64,03 | 24 605 000 |
| Ig lambda chain V-I region HA | P01700 | 12,283 | IGLV1-47 | 107,92 | 18 856 000 |
| Ig lambda chain V-I region NEW | P01701 | 12,249 | IGLV1-51 | 33,353 | 6 109 100 |
| Ig lambda chain V-I region NEWM | P01703 | 12,301 | IGLV1-40 | 71,382 | 6 959 100 |
| Ig lambda chain V-II region BOH | P01706 | 12,644 | IGLV2-11 | 37,759 | 8 440 100 |
| Ig lambda chain V-II region NEI | P01705 | 11,893 | IGLV2-23 | 4,0164 | 945 310 |
| Ig lambda chain V-II region TOG | P01704 | 12,597 | IGLV2-14 | 16,474 | 7 805 400 |
| Ig lambda chain V-III region LOI | P80748 | 12,446 | | 323,31 | 3 369 900 |
| Ig lambda chain V-III region SH | P01714 | 12,042 | IGLV3-19 | 4,7542 | 3 295 100 |
| Ig lambda chain V-IV region Kern | P01718 | 12,165 | IGLV3-27 | 20,351 | 2 645 000 |
| Ig mu chain C region | P01871 | 49,439 | IGHM | 323,31 | 779 950 000 |
| IGKV1-33 | P01602 | 12,781 | IGKV1-5 | 29,008 | 5 518 700 |
| Immunoglobulin heavy constant alpha 2 | A0A0G2JMB2 | 36,508 | IGHA2 | 323,31 | 102 440 000 |
| Immunoglobulin heavy variable 1-18 | A0A0C4DH31 | 12,82 | IGHV1-18 | 6,3184 | 4 234 400 |
| Immunoglobulin heavy variable 1-69 | P01742 | 12,66 | IGHV1-69 | 18,925 | 2 214 100 |
| Immunoglobulin heavy variable 3/OR16-9 (non-functional) | A0A0B4J2B5 | 10,657 | IGHV3OR16-9 | 235 | 53 934 000 |
| Immunoglobulin heavy variable 3-15 | A0A0B4J1V0 | 12,926 | IGHV3-15 | 31,183 | 675 050 |
| Immunoglobulin heavy variable 3-30 | P01768 | 12,965 | IGHV3-30 | 100,88 | 11 796 000 |
| Immunoglobulin heavy variable 3-72 | A0A4W8ZXM2 | 11,167 | IGHV3-72 | 18,95 | 2 742 400 |
| Immunoglobulin heavy variable 4-61 | A0A0C4DH41 | 13,016 | IGHV4-61 | 93,551 | 3 889 900 |
| Immunoglobulin heavy variable 5-51 | A0A0C4DH38 | 12,674 | IGHV5-51 | 5,8079 | 984 040 |
| Immunoglobulin J chain | P01591 | 18,098 | JCHAIN | 165,85 | 45 814 000 |

| | | | | | |
|---|---|---|---|---|---|
| Immunoglobulin kappa variable 1-12 | A0A0C4DH73 | 12,645 | IGKV1-12 | 75,525 | 10 079 000 |
| Immunoglobulin kappa variable 1-33 | P01594 | 12,848 | IGKV1-33 | 130,43 | 18 677 000 |
| Immunoglobulin kappa variable 2-24 | A0A0C4DH68 | 13,079 | IGKV2-24 | 14,382 | 869 210 |
| Immunoglobulin kappa variable 6-21 | A0A0C4DH24 | 12,43 | IGKV6-21 | 6,5648 | 1 307 700 |
| Immunoglobulin lambda constant 1 | P0CG04 | 23,063 | IGLC1 | 226,5 | 175 950 000 |
| Immunoglobulin lambda constant 3 | P0DOY3 | 11,265 | IGLC3 | 323,31 | 1 443 800 000 |
| Immunoglobulin lambda variable 2-18 | A0A075B6J9 | 12,412 | IGLV2-18 | 19,906 | 3 261 200 |
| Immunoglobulin lambda variable 3-10 | A0A075B6K4 | 12,441 | IGLV3-10 | 28,311 | 3 227 100 |
| Immunoglobulin lambda variable 8-61 | A0A075B6I0 | 12,814 | IGLV8-61 | 8,8662 | 3 019 900 |
| Insulin-like growth factor-binding protein 6 | P24592 | 25,322 | IGFBP6 | 13,425 | 1 817 600 |
| Insulin-like growth factor-binding protein complex acid labile subunit | P35858 | 66,034 | IGFALS | 205,69 | 24 559 000 |
| Inter-alpha-trypsin inhibitor heavy chain H1 | P19827 | 101,39 | ITIH1 | 323,31 | 92 483 000 |
| Inter-alpha-trypsin inhibitor heavy chain H2 | P19823 | 106,46 | ITIH2 | 323,31 | 318 710 000 |
| Inter-alpha-trypsin inhibitor heavy chain H3 | Q06033 | 99,848 | ITIH3 | 316,1 | 20 923 000 |
| Inter-alpha-trypsin inhibitor heavy chain H4 | Q14624 | 103,36 | ITIH4 | 323,31 | 233 560 000 |
| Kallistatin | P29622 | 48,541 | SERPINA4 | 168,1 | 8 801 800 |
| Kininogen-1 | P01042 | 71,957 | KNG1 | 323,31 | 506 590 000 |
| Leucine-rich alpha-2-glycoprotein | P02750 | 38,177 | LRG1 | 323,31 | 93 219 000 |
| Lipopolysaccharide-binding protein | P18428 | 53,383 | LBP | 73,031 | 5 834 300 |

| | | | | | |
|---|---|---|---|---|---|
| Low affinity immunoglobulin gamma Fc region receptor III-B | O75015 | 26,216 | FCGR3B | 6,6982 | 1 309 000 |
| L-selectin | P14151 | 42,187 | SELL | 38,497 | 2 169 800 |
| Lumican | P51884 | 38,429 | LUM | 121,45 | 15 801 000 |
| Lymphatic vessel endothelial hyaluronic acid receptor 1 | Q9Y5Y7 | 35,213 | LYVE1 | 2,5944 | 411 520 |
| Mannan-binding lectin serine protease 1 | P48740 | 79,246 | MASP1 | 11,613 | 4 007 700 |
| Monocyte differentiation antigen CD14 | P08571 | 40,076 | CD14 | 82,291 | 10 492 000 |
| Myosin-9 | P35579 | 226,53 | MYH9 | 248,28 | 5 859 100 |
| N-acetylmuramoyl-L-alanine amidase | Q96PD5 | 62,216 | PGLYRP2 | 323,31 | 87 870 000 |
| Phosphatidylcholine-sterol acyltransferase | P04180 | 49,577 | LCAT | 24,812 | 2 055 000 |
| Phosphatidylinositol-glycan-specific phospholipase D | P80108 | 92,335 | GPLD1 | 66,122 | 8 076 400 |
| Pigment epithelium-derived factor | P36955 | 46,312 | SERPINF1 | 323,31 | 41 210 000 |
| Plasma kallikrein | P03952 | 71,369 | KLKB1 | 240,26 | 34 775 000 |
| Plasma protease C1 inhibitor | P05155 | 55,154 | SERPING1 | 323,31 | 160 910 000 |
| Plasma serine protease inhibitor | P05154 | 45,674 | SERPINA5 | 45,312 | 1 290 500 |
| Plasminogen | P00747 | 90,568 | PLG | 323,31 | 298 970 000 |
| Platelet basic protein | P02775 | 21,142 | PPBP | 127,23 | 31 806 000 |
| Pregnancy zone protein | P20742 | 163,86 | PZP | 323,31 | 53 763 000 |
| Properdin | P27918 | 51,276 | CFP | 13,986 | 2 184 200 |
| Prostaglandin-H2 D-isomerase | P41222 | 21,029 | PTGDS | 13,924 | 2 309 700 |
| Protein AMBP | P02760 | 38,999 | AMBP | 323,31 | 126 750 000 |
| Protein Z-dependent protease inhibitor | Q9UK55 | 50,706 | SERPINA10 | 71,745 | 2 357 200 |
| Proteoglycan 4 | Q92954 | 151,06 | PRG4 | 24,18 | 1 975 300 |
| Prothrombin | P00734 | 70,036 | F2 | 323,31 | 365 620 000 |
| Retinol-binding protein 4 | P02753 | 23,01 | RBP4 | 323,31 | 44 838 000 |
| Selenoprotein P | P49908 | 43,173 | SEPP1 | 59,656 | 5 792 700 |
| Serotransferrin | P02787 | 77,063 | TF | 323,31 | 5 599 800 000 |

| | | | | | |
|---|---|---|---|---|---|
| Serum amyloid A-1 protein | P0DJI8 | 13,532 | SAA1 | 75,832 | 3 153 800 |
| Serum amyloid A-4 protein | P35542 | 23,353 | SAA4 | 261,98 | 31 127 000 |
| Serum amyloid P-component | P02743 | 25,387 | APCS | 52,121 | 6 237 500 |
| Serum paraoxonase/arylesterase 1 | P27169 | 39,731 | PON1 | 323,31 | 55 093 000 |
| Sex hormone-binding globulin | P04278 | 43,779 | SHBG | 102,51 | 14 790 000 |
| Talin-1 | Q9Y490 | 269,76 | TLN1 | 323,31 | 10 179 000 |
| Tetranectin | P05452 | 17,794 | CLEC3B | 24,902 | 2 168 900 |
| Thyroxine-binding globulin | P05543 | 46,324 | SERPINA7 | 153,5 | 9 615 000 |
| Transferrin receptor protein 1 | P02786 | 84,87 | TFRC | 3,6499 | 329 300 |
| Transforming growth factor-beta-induced protein ig-h3 | Q15582 | 74,68 | TGFBI | 4,8911 | 1 843 400 |
| Transthyretin | P02766 | 15,887 | TTR | 323,31 | 320 650 000 |
| Tropomyosin alpha-4 chain | P67936 | 28,521 | TPM4 | 65,228 | 3 504 700 |
| Vitamin D-binding protein | P02774 | 52,917 | GC | 323,31 | 891 350 000 |
| Vitamin K-dependent protein C | P04070 | 52,071 | PROC | 7,7852 | 1 328 400 |
| Vitamin K-dependent protein S | P07225 | 75,122 | PROS1 | 169,86 | 20 460 000 |
| Vitronectin | P04004 | 54,305 | VTN | 323,31 | 259 230 000 |
| von Willebrand factor | P04275 | 309,26 | VWF | 29,755 | 3 306 800 |
| Zinc-alpha-2-glycoprotein | P25311 | 34,258 | AZGP1 | 323,31 | 262 880 000 |

[a]**Score**: Protein score which is derived from peptide posterior error probabilities;

[b]**Intensities:** the sums of all individual peptide intensities belonging to a particular protein group.

**Table S3**
**Proteomic analysis identified 19 dysregulated proteins in genetically affected individuals with advanced CKD (stage 4 and 5) compared to genetically affected individuals with early CKD (stage 1 – 3) and genetically unaffected family members**

| Gene | Protein | Regulation (direction) | Fold change | Functional consequences | Reference | Plasma conc. Protein Atlas |
|---|---|---|---|---|---|---|
| AMBP | Alpha-1-microglobulin | Up | 2.1 | progressively increases with CKD stage | [S1, S2] | 47 mg/L |
| APOA4 | Apolipoprotein A-IV | Up | 1.9 | increases with eGFR decline | [S3, S4] | 74 mg/L |
| AZGP1 | Zinc-alpha-2-glycoprotein | Up | 1.5 | increases with ESRD | [S5, S6] | 74 mg/L |
| B2M | Beta-2-microglobulin | Up | 4.4 | tubular marker of kidney injury; the most well-characterized CKD-associated marker | [S7, S8] | 21. mg/L |
| CFD | Complement factor D | Up | 4.7 | increases in ESRD | [S2] | 4.4 mg/L |
| CD5L | CD5 antigen-like | Down | 1.9 | decreases with eGFR decline | [S3] | 4.3 mg/L |
| CST3 | Cystatin-C | Up | 4.5 | tubular marker of kidney injury; routine biomarker of ESRD | [S9] | 2.8 mg/L |
| IGFBP6 | Insulin-like growth factor-binding protein 6 | Up | 7.0 | increases in ESRD | [S2] | 300 µg/L |
| IGHM | Ig mu chain C region | Down | 2.3 | not defined for CKD/ESKD | | 110 mg/L |
| PTGDS | Prostaglandin-H2 D-isomerase | Up | 8.4 | increases in ESRD | [S2] | 2.5 mg/L |
| RBP4 | Retinol-binding protein 4 | Up | 2.2 | associated with progression of CKD; routine biomarker of ESRD | [S9, S10] | 210 mg/L |

| | | | | | | |
|---|---|---|---|---|---|---|
| SERPINF1 | Pigment epithelium-derived factor | Up | 1.9 | Increases with CKD | [S1, S2] | 44 mg/L |
| **Upregulated proteins associated with compelment, coagulation pathway and hemostasis** | | | | | | |
| **C4BPA** (C4b-binding protein alpha chain; 28 mg/L); **C4BPB** (C4b-binding protein beta chain; 1.1 mg/L); **FGG** (Fibrinogen gamma chain; 73 mg/L); **FGB** (Fibrinogen beta chain; 110 mg/L); **FGA** (Fibrinogen alpha chain; 140 mg/L); **F13B** (Coagulation factor XIII B chain; 4.7 mg/L); **CFD** (Complement factor D; 4.4 mg/L) | | | | | | |
| **Downgulated proteins associated with compelment, coagulation pathway and hemostasis** | | | | | | |
| **JCHAIN** (Immunoglobulin J chain; 2.2 mg/L) | | | | | | |

**Table S4**
**Glycoproteomic analysis identified 154 proteins**

| Protein name | Protein ID | Mol. weight [kDa] | Gene name | Score[a] | Intensities[b] |
|---|---|---|---|---|---|
| Actin, cytoplasmic 2 | P63261 | 41,792 | ACTG1 | 285,46 | 18 884 000 |
| Afamin | P43652 | 69,068 | AFM | 55,801 | 2 707 300 |
| Alpha-1-acid glycoprotein 1 | P02763 | 23,539 | ORM1 | 15,839 | 4 829 000 |
| Alpha-1-acid glycoprotein 2 | P19652 | 23,602 | ORM2 | 8,4263 | 1 223 400 |
| Alpha-1-antichymotrypsin | P01011 | 47,65 | SERPINA3 | 284,72 | 73 635 000 |
| Alpha-1-antitrypsin | P01009 | 46,736 | SERPINA1 | 323,31 | 822 210 000 |
| Alpha-1B-glycoprotein | P04217 | 54,253 | A1BG | 323,31 | 56 510 000 |
| Alpha-2-antiplasmin | P08697 | 54,565 | SERPINF2 | 137,31 | 9 748 100 |
| Alpha-2-HS-glycoprotein | P02765 | 39,34 | AHSG | 323,31 | 24 613 000 |
| Alpha-2-macroglobulin | P01023 | 163,29 | A2M | 323,31 | 872 890 000 |
| Angiotensinogen | P01019 | 52,069 | AGT | 304,75 | 23 385 000 |
| Antithrombin-III | P01008 | 52,602 | SERPINC1 | 323,31 | 96 569 000 |
| Apolipoprotein A-I | P02647 | 30,777 | APOA1 | 323,31 | 1 016 400 000 |
| Apolipoprotein A-II | P02652 | 11,175 | APOA2 | 114,29 | 43 922 000 |
| Apolipoprotein A-IV | P06727 | 45,371 | APOA4 | 323,31 | 73 898 000 |
| Apolipoprotein B-100 | P04114 | 515,6 | APOB | 323,31 | 342 600 000 |
| Apolipoprotein C-I | P02654 | 8,647 | APOC1 | 3,0138 | 751 830 |
| Apolipoprotein C-II | P02655 | 11,284 | APOC2 | 2,6927 | 644 350 |
| Apolipoprotein C-III | P02656 | 10,852 | APOC3 | 53,129 | 6 104 700 |
| Apolipoprotein D | P05090 | 24,158 | APOD | 117,44 | 3 719 300 |
| Apolipoprotein E | P02649 | 36,154 | APOE | 323,31 | 40 181 000 |
| Apolipoprotein L1 | O14791 | 43,974 | APOL1 | 180,02 | 8 062 400 |
| Apolipoprotein(a) | P08519 | 226,54 | LPA | 305,59 | 44 504 000 |
| Beta-2-glycoprotein 1 | P02749 | 38,298 | APOH | 323,31 | 35 153 000 |
| C4b-binding protein alpha chain | P04003 | 67,033 | C4BPA | 323,31 | 105 830 000 |
| C4b-binding protein beta chain | P20851 | 28,357 | C4BPB | 56,932 | 6 963 000 |
| Carboxypeptidase B2 | Q96IY4 | 48,424 | CPB2 | 17,243 | 1 165 700 |
| Carboxypeptidase N catalytic chain | P15169 | 52,286 | CPN1 | 10,011 | 745 450 |
| Carboxypeptidase N subunit 2 | P22792 | 60,556 | CPN2 | 46,205 | 4 886 800 |
| CD5 antigen-like | O43866 | 38,087 | CD5L | 323,31 | 30 310 000 |
| Ceruloplasmin | P00450 | 122,2 | CP | 323,31 | 117 930 000 |
| Clusterin | P10909 | 52,494 | CLU | 323,31 | 95 113 000 |
| Coagulation factor IX | P00740 | 51,778 | F9 | 29,67 | 1 682 800 |
| Coagulation factor V | P12259 | 251,7 | F5 | 13,696 | 430 490 |
| Coagulation factor X | P00742 | 54,731 | F10 | 4,9505 | 2 763 000 |
| Coagulation factor XII | P00748 | 67,791 | F12 | 323,31 | 14 214 000 |
| Coagulation factor XIII A chain | P00488 | 83,267 | F13A1 | 7,5129 | 490 000 |
| Coagulation factor XIII B chain | P05160 | 75,51 | F13B | 119,72 | 5 546 400 |

| | | | | | |
|---|---|---|---|---|---|
| Complement C1q subcomponent subunit A | P02745 | 26,016 | C1QA | 69,641 | 4 211 900 |
| Complement C1q subcomponent subunit B | P02746 | 26,721 | C1QB | 114,86 | 7 426 700 |
| Complement C1q subcomponent subunit C | P02747 | 25,773 | C1QC | 34,358 | 15 463 000 |
| Complement C1r subcomponent | P00736 | 80,118 | C1R | 209,42 | 12 862 000 |
| Complement C1s subcomponent | P09871 | 76,684 | C1S | 129,16 | 21 862 000 |
| Complement C2 | B4E1Z4 | 140,94 | CFB | 323,31 | 217 920 000 |
| Complement C2 | P06681 | 83,267 | C2 | 10,836 | 1 703 200 |
| Complement C3 | P01024 | 187,15 | C3 | 323,31 | 1 098 200 000 |
| Complement C4-A | P0C0L4 | 192,78 | C4A | 25,328 | 10 605 000 |
| Complement C4-B | P0C0L5 | 192,75 | C4B | 323,31 | 381 750 000 |
| Complement C5 | P01031 | 188,3 | C5 | 154,53 | 15 585 000 |
| Complement component C6 | P13671 | 104,79 | C6 | 269,88 | 16 266 000 |
| Complement component C7 | P10643 | 93,517 | C7 | 180,41 | 11 398 000 |
| Complement component C8 alpha chain | P07357 | 65,163 | C8A | 245,38 | 13 395 000 |
| Complement component C8 beta chain | P07358 | 67,046 | C8B | 81,722 | 6 530 800 |
| Complement component C8 gamma chain | P07360 | 22,277 | C8G | 186,78 | 9 423 300 |
| Complement component C9 | P02748 | 63,173 | C9 | 201,04 | 30 927 000 |
| Complement factor D | P00746 | 27,033 | CFD | 61,406 | 869 460 |
| Complement factor H | Q5TFM2 | 51,007 | CFH | 39,764 | 838 920 |
| Complement factor H | P08603 | 139,09 | CFH | 323,31 | 325 200 000 |
| Complement factor H-related protein 1 | Q03591 | 37,65 | CFHR1 | 131,56 | 5 841 900 |
| Complement factor H-related protein 2 | V9GYE7 | 28,912 | CFHR2 | 4,9143 | 1 063 200 |
| Complement factor H-related protein 4 | Q92496 | 65,35 | CFHR4 | 5,5033 | 625 460 |
| Complement factor I | P05156 | 65,75 | CFI | 295,19 | 36 325 000 |
| Corticosteroid-binding globulin | P08185 | 45,14 | SERPINA6 | 112,99 | 7 767 600 |
| EGF-containing fibulin-like extracellular matrix protein 1 | Q12805 | 54,64 | EFEMP1 | 29,192 | 278 530 |
| Fibrinogen alpha chain | P02671 | 94,972 | FGA | 323,31 | 1 306 800 000 |
| Fibrinogen beta chain | P02675 | 55,928 | FGB | 323,31 | 980 990 000 |
| Fibrinogen gamma chain | P02679 | 51,511 | FGG | 323,31 | 590 820 000 |
| Fibronectin | P02751 | 272,32 | FN1 | 323,31 | 132 310 000 |
| Fibulin-1 | B1AHL2 | 78,329 | FBLN1 | 57,813 | 3 297 300 |
| Filamin-A | P21333 | 280,74 | FLNA | 323,31 | 8 259 600 |

| | | | | | |
|---|---|---|---|---|---|
| Galectin-3-binding protein | Q08380 | 65,33 | LGALS3BP | 51,874 | 1 750 400 |
| Gelsolin | P06396 | 85,696 | GSN | 323,31 | 69 678 000 |
| Haptoglobin | P00738 | 45,205 | HP | 323,31 | 869 700 000 |
| Haptoglobin-related protein | P00739 | 39,029 | HPR | 156,28 | 7 495 100 |
| Heat shock cognate 71 kDa protein | P11142 | 70,897 | HSPA8 | 122,3 | 3 292 100 |
| Hemoglobin subunit alpha | P69905 | 15,257 | HBA1 | 268,39 | 41 766 000 |
| Hemoglobin subunit beta | P68871 | 15,998 | HBB | 323,31 | 68 331 000 |
| Hemopexin | P02790 | 51,676 | HPX | 323,31 | 264 630 000 |
| Heparin cofactor 2 | P05546 | 57,07 | SERPIND1 | 79,624 | 15 116 000 |
| Hepatocyte growth factor-like protein | P26927 | 80,319 | MST1 | 5,4518 | 445 920 |
| Histidine-rich glycoprotein | P04196 | 59,578 | HRG | 276,58 | 80 286 000 |
| Hyaluronan-binding protein 2 | Q14520 | 62,671 | HABP2 | 43,154 | 5 632 700 |
| Ig alpha-1 chain C region | P01876 | 37,654 | IGHA1 | 323,31 | 735 380 000 |
| Ig delta chain C region | P01880 | 42,352 | IGHD | 51,894 | 3 590 100 |
| Ig gamma-1 chain C region | P01857 | 36,105 | IGHG1 | 323,31 | 474 340 000 |
| Ig gamma-2 chain C region | P01859 | 35,9 | IGHG2 | 323,31 | 264 290 000 |
| Ig gamma-3 chain C region | P01860 | 41,287 | IGHG3 | 323,31 | 649 030 000 |
| Ig gamma-4 chain C region | P01861 | 35,94 | IGHG4 | 290,3 | 70 040 000 |
| Ig heavy chain V-I region V35 | P23083 | 13,085 | | 11,29 | 2 157 500 |
| Ig heavy chain V-III region DOB | P01782 | 12,945 | IGHV3-9 | 109,49 | 2 526 600 |
| Ig kappa chain C region | P01834 | 11,765 | IGKC | 323,31 | 1 180 700 000 |
| Ig kappa chain V-I region Gal | P01599 | 12,778 | IGKV1-17 | 10,604 | 955 480 |
| Ig kappa chain V-II region RPMI 6410 | P06310 | 13,185 | | 11,887 | 2 415 100 |
| Ig kappa chain V-III region B6 | P01619 | 12,557 | IGKV3-20 | 303,9 | 28 638 000 |
| Ig kappa chain V-III region POM | P01624 | 12,496 | IGKV3-15 | 51,792 | 17 273 000 |
| Ig kappa chain V-IV region | P06312 | 13,38 | IGKV4-1 | 23,466 | 4 312 300 |
| Ig lambda chain V-I region HA | P01700 | 12,283 | IGLV1-47 | 67,344 | 10 643 000 |
| Ig lambda chain V-I region NEW | P01701 | 12,249 | IGLV1-51 | 4,4745 | 1 948 200 |
| Ig lambda chain V-I region NEWM | P01703 | 12,301 | IGLV1-40 | 2,0244 | 495 600 |
| Ig lambda chain V-II region MGC | P01709 | 12,382 | IGLV2-8 | 8,3972 | 3 406 500 |
| Ig lambda chain V-IV region Hil | P01717 | 12,011 | IGLV3-25 | 5,5346 | 1 750 400 |
| Ig mu chain C region | P01871 | 49,439 | IGHM | 323,31 | 430 080 000 |
| Ig-like domain-containing protein | A0A0G2JRQ6 | 12,748 | | 2,4192 | 2 818 300 |
| Ig-like domain-containing protein | A0A0J9YY99 | 12,965 | IGHV3-23 | 26,688 | 5 150 200 |
| Immunoglobulin heavy constant alpha 2 | A0A0G2JMB2 | 36,508 | IGHA2 | 177,16 | 20 298 000 |

| | | | | | |
|---|---|---|---|---|---|
| Immunoglobulin heavy variable 1-18 | A0A0C4DH31 | 12,82 | IGHV1-18 | 4,4334 | 673 940 |
| Immunoglobulin heavy variable 3/OR16-9 (non-functional) | A0A0B4J2B5 | 10,657 | IGHV3OR16-9 | 106,17 | 34 934 000 |
| Immunoglobulin heavy variable 3-49 | A0A0A0MS15 | 13,056 | IGHV3-49 | 6,9507 | 796 860 |
| Immunoglobulin heavy variable 3-72 | A0A4W8ZXM2 | 11,167 | IGHV3-72 | 21,125 | 3 677 800 |
| Immunoglobulin heavy variable 3-74 | A0A0B4J1X5 | 12,839 | IGHV3-74 | 54,963 | 14 022 000 |
| Immunoglobulin heavy variable 4-38-2 | P0DP08 | 13,016 | IGHV4-61 | 114,6 | 3 821 300 |
| Immunoglobulin heavy variable 5-51 | A0A0C4DH38 | 12,674 | IGHV5-51 | 22,386 | 1 692 300 |
| Immunoglobulin J chain | P01591 | 18,098 | JCHAIN | 173,46 | 39 086 000 |
| Immunoglobulin kappa variable 2-24 | A0A0C4DH68 | 13,079 | IGKV2-24; | 32,2 | 2 543 100 |
| Immunoglobulin kappa variable 2D-40 | P01614 | 13,31 | IGKV2D-40 | 50,848 | 17 851 000 |
| Immunoglobulin kappa variable 3D-11 | A0A0A0MRZ8 | 12,625 | IGKV3D-11 | 44,645 | 19 429 000 |
| Immunoglobulin kappa variable 6D-21 | A0A0A0MT36 | 12,34 | IGKV6D-21 | 19,968 | 560 790 |
| Immunoglobulin lambda constant 3 | P0DOY3 | 11,265 | | 323,31 | 490 760 000 |
| Immunoglobulin lambda variable 3-10 | A0A075B6K4 | 12,441 | IGLV3-10 | 13,728 | 9 241 900 |
| Immunoglobulin lambda variable 3-9 | A0A075B6K5 | 12,332 | IGLV3-9 | 182,35 | 17 076 000 |
| Immunoglobulin lambda variable 8-61 | A0A075B6I0 | 12,814 | IGLV8-61 | 2,1839 | 2 614 700 |
| Immunoglobulin lambda-like polypeptide 5 | B9A064 | 23,063 | IGLL5 | 66,658 | 90 516 000 |
| Insulin-like growth factor-binding protein complex acid labile subunit | P35858 | 66,034 | IGFALS | 63,479 | 4 357 000 |
| Inter-alpha-trypsin inhibitor heavy chain H1 | P19827 | 101,39 | ITIH1 | 323,31 | 77 724 000 |
| Inter-alpha-trypsin inhibitor heavy chain H2 | P19823 | 106,46 | ITIH2 | 323,31 | 156 040 000 |
| Inter-alpha-trypsin inhibitor heavy chain H3 | Q06033 | 99,848 | ITIH3 | 83,282 | 7 083 000 |
| Inter-alpha-trypsin inhibitor heavy chain H4 | Q14624 | 103,36 | ITIH4 | 323,31 | 92 477 000 |
| Kallistatin | P29622 | 48,541 | SERPINA4 | 4,1079 | 2 584 000 |

| | | | | | |
|---|---|---|---|---|---|
| Kininogen-1 | P01042 | 71,957 | KNG1 | 323,31 | 104 870 000 |
| Leucine-rich alpha-2-glycoprotein | P02750 | 38,177 | LRG1 | 207,91 | 16 424 000 |
| Lumican | P51884 | 38,429 | LUM | 59,504 | 2 928 700 |
| N-acetylmuramoyl-L-alanine amidase | Q96PD5 | 62,216 | PGLYRP2 | 138,68 | 5 083 800 |
| Phosphatidylinositol-glycan-specific phospholipase D | P80108 | 92,335 | GPLD1 | 15,266 | 2 031 200 |
| Pigment epithelium-derived factor | P36955 | 46,312 | SERPINF1 | 309,58 | 17 906 000 |
| Plasma kallikrein | P03952 | 71,369 | KLKB1 | 272,56 | 19 496 000 |
| Plasma protease C1 inhibitor | P05155 | 55,154 | SERPING1 | 124,67 | 22 195 000 |
| Plasminogen | P00747 | 90,568 | PLG | 323,31 | 143 890 000 |
| Pregnancy zone protein | P20742 | 163,86 | PZP | 323,31 | 38 742 000 |
| Properdin | P27918 | 51,276 | CFP | 41,291 | 1 901 500 |
| Protein AMBP | P02760 | 38,999 | AMBP | 280,43 | 70 170 000 |
| Protein Z-dependent protease inhibitor | Q9UK55 | 50,706 | SERPINA10 | 27,001 | 365 530 |
| Prothrombin | P00734 | 70,036 | F2 | 323,31 | 99 495 000 |
| Retinol-binding protein 4 | P02753 | 23,01 | RBP4 | 9,7668 | 1 408 100 |
| Selenoprotein P | P49908 | 43,173 | SEPP1 | 3,9702 | 669 150 |
| Serotransferrin | P02787 | 77,063 | TF | 323,31 | 2 380 500 000 |
| Serum amyloid A-4 protein | A0A096LPE2 | 23,353 | SAA2-SAA4 | 48,344 | 12 689 000 |
| Serum amyloid P-component | P02743 | 25,387 | APCS | 21,71 | 1 074 200 |
| Serum paraoxonase/arylesterase 1 | P27169 | 39,731 | PON1 | 323,31 | 30 521 000 |
| Tetranectin | P05452 | 17,794 | CLEC3B | 14,99 | 1 742 000 |
| Thyroxine-binding globulin | P05543 | 46,324 | SERPINA7 | 55,761 | 2 797 400 |
| Transthyretin | P02766 | 15,887 | TTR | 323,31 | 119 510 000 |
| Vitamin D-binding protein | P02774 | 52,917 | GC | 323,31 | 113 200 000 |
| Vitamin K-dependent protein S | P07225 | 75,122 | PROS1 | 193,91 | 12 836 000 |
| Vitronectin | P04004 | 54,305 | VTN | 268,63 | 136 540 000 |
| von Willebrand factor | P04275 | 309,26 | VWF | 16,888 | 514 330 |

[a]**Score**: Protein score which is derived from peptide posterior error probabilities;

[b]**Intensities**: the sums of all individual peptide intensities belonging to a particular protein group.

**Table S5**

**Glycoproteomic analysis identified 12 dysregulated proteins in genetically affected individuals with advanced CKD (stage 4 and 5) compared to genetically affected individuals with early CKD (stage 1 – 3) and genetically unaffected family members.**

| Gene | Protein | Regulation (direction) | Detected as significant in proteomics | Fold change | Functional consequences | Reference | Plasma conc. Protein Atlas |
|---|---|---|---|---|---|---|---|
| AMBP | Alpha-1-microglobulin | UP | Y | 1.7 | progressively increases in plasma with increasing of CKD stage: from CKD1-2 to CKD3-4 and finally to CKD5 | [S1, S2] | 47 mg/L |
| APOA4 | Apolipoprotein A-IV | UP | Y | 2.8 | increases is associated with eGFR decline | [S3, S4] | 74 mg/L |
| CD5L | CD5 antigen-like | DOWN | Y | 2.1 | decreases is associated with eGFR decline | [S3] | 4.3 mg/L |
| CFD | Complement factor D | UP | Y | 5.3 | increases in ESRD; complement and coagulation pathway; hemostasis (DAVID) | [S2] | 4.4 mg/L |
| HP | Haptoglobin | UP | N | 1.9 | acute phase reactant | [S11] | 100 mg/L |
| HSPA8 | Heat shock cognate 71 kDa protein | UP | N | 4.7 | upregulated in activated stress of endoplasmic reticulum | [S12] | 140 μg/L |
| IGHM | Ig mu chain C region | DOWN | Y | 2.7 | not defined for CKD/ESKD | | 110 mg/L |
| KNG1 | Kininogen-1 | UP | N | 1.3 | increases in renal function decline | [S13] | 47 mg/L |
| LRG1 | Leucine-rich alpha-2-glycoprotein | UP | N | 1.7 | involved in kidney disease progression | [S14] | 42 mg/L |
| LUM | Lumican | UP | N | 2.0 | together with fibromodulin is involved in renal fibrosis | [S15] | 29 mg/L |
| RBP4 | Retinol-binding protein 4 | UP | Y | 2.8 | associated to the progression of renal failure and end-stage renal, routine biomarker of ESRD | [S9, S10, S16] | 210 mg/L |
| SERPINF1 | Pigment epithelium-derived factor | UP | Y | 1.9 | increases is associated with CKD | [S1, S2] | 44 mg/L |

## Supplementary Methods

### 2.1 Subjects and Study Approval

A non-consanguineous kindred was referred to our genetic service at the Irish Kidney Gene Project. All individuals provided informed consent. The study was approved by the Ethical Committee of Beaumont Hospital (REC 19/28). Approval for the release of biopsy material was also obtained.

Clinical information, including age at first clinical presentation, serum creatinine values, and imaging findings, was obtained through chart review or clinic visits. Hypertension was defined by blood pressure above 140/90 mmHg and/or the prescription of antihypertensive medications. CKD was defined as an estimated glomerular filtration rate (eGFR) less than 60 ml/min/1.73 m2 for 3 months or longer, according to the CKD-EPI (Chronic Kidney Disease Epidemiology Collaboration 2021) equation [S17]. Screening for proteinuria was assessed using the spot urine protein/creatinine ratio (mg/mmol). The commencement of kidney replacement therapy or pre-emptive kidney transplant was defined as ESKD. Ultrasound was used to assess kidney and liver size, echogenicity, and the presence of cysts. If unavailable, prior imaging studies were examined. An individual was considered to be affected if one of the following clinical characteristics was present: multiple kidney cysts, non-enlarged kidneys with nephronophthisis-like histological characteristics, or CKD.

### 2.2 Genetic Analysis

Genomic DNA was extracted from blood using standard procedures. Genetic testing included Sanger sequencing of UMOD [S18], and REN [S19], targeted genotyping [S20] and long-read sequencing of MUC1 [S21], 227-genes panel [S22, S23], whole-exome sequencing, and targeted ALG5 variant genotyping. Data analysis, variant prioritization, and targeted genotyping were performed as previously described [S24]. PLINK 1.9 was used to estimate genome-wide identity by descent (IBD)-sharing coefficients between members of both families (n=3) to determine whether the ALG5 variant is of a common descent [S25]. Using variants from the WES data imported to PLINK with the --vcf command, relatedness measures were inferred, and coefficients were calculated using --genome **(Supplementary Table S1).**

### 2.3 In-Silico Analysis

For structural mapping, the AlphaFold model of human dolichyl-phosphate beta-glucosyltransferase activity was used (AF-Q9Y673-F1). Regions involved in enzymatic catalysis were determined from archeal dolichyl-phosphate mannose synthase crystal structures reported previously [S26]. Structural models were visualized using Pymol and ChimeraX.

### 2.4 Histopathological Staining and Immunohistochemical Detection of ALG5 in Kidney Biopsy

Formaldehyde-fixed paraffin-embedded kidney biopsies were available in three affected subjects as part of their clinical workup. Masson's trichrome and hematoxylin-eosin staining was carried out according to standard protocols on 5-µm-thick kidney sections. Biopsy specimens were stained for immunofluorescence studies using commercially available antisera. Electron microscopy was performed following standard protocols.

### 2.5 Immunofluorescence Analysis of Human Kidney Biopsy

Formaldehyde-fixed paraffin-embedded kidney biopsies were obtained from three patients and two control samples. Control samples were examined by a renal pathologist to select healthy tissue for further processing. For ALG5 patient tissues, samples from the kidney cortex and medulla were collected from patients with advanced disease.

The paraffin sections were stained after deparaffination, hydration, sodium citrate pretreatment (pH=6), and standard blocking procedures (blocking of endogenous peroxidase with 1% sodium azide and 0.3% H2O2 for 10 minutes and blocking with 5% fetal bovine serum (FBS) in phosphate-buffered saline (PBS) for 30 minutes, both at room temperature).

Immunohistochemical detection of ALG5 was achieved with Rabbit polyclonal anti-human ALG5 antibody (Novusbio, NBP2-92371) diluted 1:100 in PBS. The bound primary antibody was detected with Dako EnVision+TM Peroxidase Rabbit Kit (Dako, Glostrup, Denmark).

For parallel immunofluorescence detection of either ALG5 or UMOD with ER, ER-Golgi intermediate compartment (ERGIC), Golgi apparatus or plasma membrane markers, kidney sections were incubated overnight at 4°C with following antibodies: ALG5 with polyclonal rabbit anti-ALG5 antibody (Novus Biologicals) diluted 1:200; UMOD with polyclonal sheep anti-THP/UMOD antibody (Tamm-Horsfall Glycoprotein (THG) antibody, My BioSource) diluted 1:300; ER with monoclonal mouse anti-PDI antibody (ENZO Life Sciences) diluted 1:50; ERGIC with monoclonal mouse anti-LMAN1 antibody (Thermo Fisher Scientific) diluted 1:100; Golgi apparatus with monoclonal mouse anti-58K antibody (Abcam) diluted 1:50 and plasma membrane with rabbit anti-pan Cadherin (Thermo Fisher Scientific) diluted 1:50 in 5% BSA in PBS. Detection of bound primary antibodies was achieved using Donkey anti-Mouse IgG Alexa Fluor®647, Donkey anti-Rabbit IgG Alexa Fluor®488 and Donkey anti-Sheep IgG Alexa Fluor®555 secondary antibodies (Thermo Fischer Scientific) diluted 1:500 in 5% BSA in PBS.

### 2.6 Image Acquisition and Analysis

XYZ images were sampled according to Nyquist criterion using a Leica SP8X laser scanning confocal microscope, HC PL Apo objective (633, N.A.1.40), 405 nm diode/50 mW DMOD Flexibl, and 488, 555, and 647 laser lines in 470–670 nm 80 MHz pulse continuum WLL2. Images were restored using a classic maximum likelihood restoration algorithm in the Huygens Professional Software (SVI, Hilversum, The Netherlands) [S27]. The colocalization maps employing single pixel overlap coefficient values ranging from 0-1 were created in the Huygens Professional Software [S28]. The resulting overlap coefficient values are presented as the pseudo color, which scale is shown in corresponding lookup tables (LUT).

### 2.7 Western Blot Analysis of Urinary Uromodulin

Aliquots of spot urines were normalized to urinary creatinine concentration, denatured in 6x Sodium dodecyl sulfate-polyacrylamide gel electrophoresis (SDS-PAGE) loading buffer at 100 °C for 5 minutes, and resolved on 10% Mini-PROTEAN® TGX™ Precast Protein Gel (Bio-Rad) in Mini-Protean 3 Cell with SDS-PAGE running buffer (191.8 mM Glycine, 234.8 mM Tris base, 3.47 mM SDS). Proteins were transferred onto Immobilon-E PVDF membrane (Merck Millipore Ltd.) using Trans-Blot Turbo Transfer System (Bio-Rad) with Bjerrum Schafer-Nielsen buffer

with SDS. After membrane drying, reactivation in pure methanol for 20 s and blocking in 2% Bovine Serum Albumin (BSA) (#11930.04, Serva) for 2 h, uromodulin was detected with polyclonal sheep Tamm-Horsfall Glycoprotein (THG) antibody (#MBS535021, MyBiosource) diluted 1:5000 and rabbit anti-sheep IgG (H+L)–Peroxidase antibody (#81-8620, Thermo Fisher Scientific) diluted 1:10000 in 1x PBS-Tween (0.1 %) with 2% BSA (1 h each). After final washes in 1x PBS-T and PBS, membranes were developed in a 1:1 mixture of Clarity Western ECL Substrate (Bio-Rad) solutions for 5 minutes. The chemiluminescent signal was recorded by ChemiDoc MP Imaging System (Bio-Rad). All antibody incubations and washes were performed at room temperature.

**2.8 Plasma measurements**

Plasma uromodulin concentration was determined by the Uromodulin Human ELISA kit (Biovendor, Czech Republic) according to manufacturer instructions. Plates were washed in ELx50 automatic plate washer (Biotek Instruments, USA) and read in SLT Spectra plate reader (SLT Labinstruments GmbH, Austria). Measured data were processed by KIM Immunochemical Processing software (Daniel Kittrich, Czech Republic).

Plasma CA15-3 (mucin 1) concentration was determined as previously described [S29].

**2.9 Western Blot Analysis of Plasma Transferrin**

Electrophoresis and western blot of plasma transferrin was performed according to Seta et al [S30] with minor modifications. Plasma sample was diluted 20x in dH2O and then 100x in sample buffer (10 mM TRIS/HCl, pH 8, 1 mM EDTA, 2,5% (v/v) glycerol, 0,05% (w/V) Bromphenol blue, 2,5 % SDS (w/V)). 10 ul of denaturated sample was loaded to the 4% separating gel. SDS-PAGE was carried under standard conditions with 12% polyacrylamide, 0.1% (w/v) SDS gels and glycine running buffer (25 mM TRIS/HCl, 250 mM Glycine, 0,1% SDS (w/V)). Specific protein bands were detected with 1:1000 dilution of rabbit anti-human transferrin antibody (Dako, Glostrub, Denmark) and the signal was quantified using Quantity one software (Bio-Rad Laboratories, Hercules, California).

**2.10 Proteomic and Glycoproteomic Profiles of Plasma**

Plasma protein concentration was determined using a BCA Protein Assay Kit (ThermoScientific). From each sample, an aliquot equivalent to 100 µg of protein was taken and divided into two parts. One part was subjected directly to the proteomic analysis, while the other one underwent an albumin depletion protocol, as detailed in [S31], to enrich glycoprotein content. Briefly, after sample dilution with water, the lipid fraction was removed. Then, 150 mM NaCl and ice-cold ethanol were added to the samples in a final ratio of 1:2:2.3, respectively. The samples were incubated for 1 hour at 4°C and then centrifuged at 4°C and 16,000 × g for 45 minutes. The resulting pellet was resuspended in 42% ice-cold ethanol and centrifuged at 4°C and 16,000 × g for an additional 15 minutes. The final pellet obtained after these steps was used for proteomic analysis.

Proteomic analysis was carried out using a sample aliquot containing 20 µg of protein. To each sample, a 100 mM triethylammonium bicarbonate buffer (TEAB) was added to adjust the total volume to 40 µL. Next, the samples were reduced and alkylated with tris(2-

carboxyethyl)phosphine (TCEP) at a concentration of 10 mM and 2-chloroacetamide (CAA) at a concentration of 50 mM. The samples were then incubated at 70°C for 5 minutes. For digestion, trypsin was added to the samples at a ratio of 1:20, and the mixture was kept at 37°C overnight. To terminate the digestion process, trifluoroacetic acid (TFA) was added to achieve a final concentration of 0.5%.

After the protein digestion, the sample was subjected to offline desalting using C18 StageTip (3M, USA) [S32]. Following desalting, the sample was dried using a SpeedVac concentrator. The dried peptides were re-suspended in a solution containing 2% acetonitrile (ACN) and 0.1% trifluoroacetic acid (TFA). The analysis of the peptides was performed using the Vanquish liquid chromatography system (ThermoScientific), which was coupled to the timsToF SCP mass spectrometer equipped with Captive spray (Bruker Daltonics). The mass spectrometer operated in a positive data-dependent mode. A one-microliter volume of the peptide mixture was injected using an autosampler onto a C18 trap column (PepMap Neo C18 5µm, 300 µm × 5 mm, Thermo Scientific). After 3 minutes of trapping, the peptides were eluted from the trap column and separated on a C18 analytical column (DNV PepMap Neo 75 µm × 150 mm, 2 µm, Thermo Scientific) using a linear gradient of 5% to 35% ACN over 35 minutes at a flow rate of 350 nL/min. Both the trap and analytical columns were heated to 50°C. The timsTOF SCP settings were based on the standard proteomics PASEF method. The scan range was set from 0.6 to 1.6 V s/cm2 with a ramp time of 100 ms. A total of 10 PASEF MS/MS scans were performed. Precursor ions in the m/z range between 100 and 1700 with charge states ≥2+ and ≤6+ were selected for fragmentation. Active exclusion was enabled for 0.4 min to prevent repeated selection of the same precursor ions.

Proteins were identified using MaxQuant software (version 1.6.17) [S33], and the peak lists were searched against the Homo sapiens database from Uniprot using the Andromeda search engine [S34]. The database search parameters were as follows: trypsin enzyme specificity, allowing up to two missed cleavages; fixed modifications included carbamidomethylation of cysteine, while variable modifications were N-terminal protein acetylation and methionine oxidation. The precursor ion tolerance was set at 20 ppm, and the mass tolerance for MS/MS fragment ions was set at 0.5 Da. To ensure reliable identifications, PSM (Peptide Spectrum Match) and protein identifications were filtered using a target-decoy approach at a false discovery rate (FDR) of 1%.

For label-free quantification (LFQ) of proteins, the MaxLFQ algorithm [S35] integrated into MaxQuant was utilized. A minimum ratio count of 2 was set to ensure robust quantification. Subsequent data analysis was performed using Perseus software (version 2.0.70) [S36]. The data were filtered to remove hits to the reverse database, contaminants, and proteins identified solely with modified peptides. LFQ intensity values were log2-transformed and normalized using Z-score. Statistical analyses were carried out using algorithms integrated into Perseus.

The raw data were deposited to a ProteomeXchange Consortium via a PRIDE [S37] partner repository with a data set identifier**.**

**2.11 Statistical analysis**

Patient characteristics and genetic diagnosis were collected, and data were presented descriptively; continuous variables were expressed as mean ± standard deviation or median [interquartile range, IQR], whereas categorical variables were expressed in frequencies or percentages. The difference in means between subgroups was determined using a two-sample Student t-test, with a two-tailed P value of less than 0.05 indicating statistical significance. Kaplan-Meier curves with log-rank testing were used to evaluate progression to end-stage kidney disease. Statistical analyses were performed using STATA SE 16 (StataCorp, College Station, TX, USA).

STROBE Statement—checklist of items that should be included in reports of observational studies

**A Novel Monoallelic *ALG5* Variant Causing Late-onset ADPKD and Tubulointerstitial Fibrosis**

| | Item No. | Recommendation | Page No. | Relevant text from manuscript |
|---|---|---|---|---|
| **Title and abstract** | 1 | (*a*) Indicate the study's design with a commonly used term in the title or the abstract | 1 | |
| | | (*b*) Provide in the abstract an informative and balanced summary of what was done and what was found | 3 | |
| **Introduction** | | | | |
| Background/rationale | 2 | Explain the scientific background and rationale for the investigation being reported | 5 | |
| Objectives | 3 | State specific objectives, including any prespecified hypotheses | 5 | |
| **Methods** | | | | |
| Study design | 4 | Present key elements of study design early in the paper | 6 | |
| Setting | 5 | Describe the setting, locations, and relevant dates, including periods of recruitment, exposure, follow-up, and data collection | 6 | |
| Participants | 6 | (*a*) *Cohort study*—Give the eligibility criteria, and the sources and methods of selection of participants. Describe methods of follow-up<br>*Case-control study*—Give the eligibility criteria, and the sources and methods of case ascertainment and control selection. Give the rationale for the choice of cases and controls<br>*Cross-sectional study*—Give the eligibility criteria, and the sources and methods of selection of participants | 6, supplemental methods 2.1 Cross-sectional study | |
| | | (*b*) *Cohort study*—For matched studies, give matching criteria and number of exposed and unexposed<br>*Case-control study*—For matched studies, give matching criteria and the number of controls per case | NA | |

| | | | |
|---|---|---|---|
| Variables | 7 | Clearly define all outcomes, exposures, predictors, potential confounders, and effect modifiers. Give diagnostic criteria, if applicable | 10, Supplementary Methods 2.10 |
| Data sources/ measurement | 8* | For each variable of interest, give sources of data and details of methods of assessment (measurement). Describe comparability of assessment methods if there is more than one group | 10, Supplementary Methods 2.10 |
| Bias | 9 | Describe any efforts to address potential sources of bias | NA |
| Study size | 10 | Explain how the study size was arrived at | 6, Supplementary Methods 2.1 |

Continued on next page

| | | | |
|---|---|---|---|
| Quantitative variables | 11 | Explain how quantitative variables were handled in the analyses. If applicable, describe which groupings were chosen and why | 10 |
| Statistical methods | 12 | (*a*) Describe all statistical methods, including those used to control for confounding | 10, Supplementary Methods 2.10 |
| | | (*b*) Describe any methods used to examine subgroups and interactions | NA |
| | | (*c*) Explain how missing data were addressed | NA |
| | | (*d*) *Cohort study*—If applicable, explain how loss to follow-up was addressed<br>*Case-control study*—If applicable, explain how matching of cases and controls was addressed<br>*Cross-sectional study*—If applicable, describe analytical methods taking account of sampling strategy | NA |
| | | (*e*) Describe any sensitivity analyses | NA |
| **Results** | | | |
| Participants | 13* | (a) Report numbers of individuals at each stage of study—eg numbers potentially eligible, examined for eligibility, confirmed eligible, included in the study, completing follow-up, and analysed | 11-12 |
| | | (b) Give reasons for non-participation at each stage | NA |
| | | (c) Consider use of a flow diagram | Pedigrees Figures 1 and 2 |
| Descriptive data | 14* | (a) Give characteristics of study participants (eg demographic, clinical, social) and information on exposures and potential confounders | 12 |
| | | (b) Indicate number of participants with missing data for each variable of interest | NA |
| | | (c) *Cohort study*—Summarise follow-up time (eg, average and total amount) | NA |
| Outcome data | 15* | *Cohort study*—Report numbers of outcome events or summary measures over time | NA |
| | | *Case-control study*—Report numbers in each exposure category, or summary measures of exposure | NA |
| | | *Cross-sectional study*—Report numbers of outcome events or summary measures | 11, 12, 13, 14-15 |
| Main results | 16 | (*a*) Give unadjusted estimates and, if applicable, confounder-adjusted estimates and their precision (eg, 95% confidence interval). Make clear which confounders were adjusted for and why they were included | NA |
| | | (*b*) Report category boundaries when continuous variables were categorized | NA |
| | | (*c*) If relevant, consider translating estimates of relative risk into absolute risk for a meaningful time period | NA |

Continued on next page

| | | | |
|---|---|---|---|
| Other analyses | 17 | Report other analyses done—eg analyses of subgroups and interactions, and sensitivity analyses | NA |
| **Discussion** | | | |
| Key results | 18 | Summarise key results with reference to study objectives | 15-18 |
| Limitations | 19 | Discuss limitations of the study, taking into account sources of potential bias or imprecision. Discuss both direction and magnitude of any potential bias | 18 |
| Interpretation | 20 | Give a cautious overall interpretation of results considering objectives, limitations, multiplicity of analyses, results from similar studies, and other relevant evidence | 15-18 |
| Generalisability | 21 | Discuss the generalisability (external validity) of the study results | 18 |
| **Other information** | | | |
| Funding | 22 | Give the source of funding and the role of the funders for the present study and, if applicable, for the original study on which the present article is based | 19-20 |

*Give information separately for cases and controls in case-control studies and, if applicable, for exposed and unexposed groups in cohort and cross-sectional studies.

**Note:** An Explanation and Elaboration article discusses each checklist item and gives methodological background and published examples of transparent reporting. The STROBE checklist is best used in conjunction with this article (freely available on the Web sites of PLoS Medicine at http://www.plosmedicine.org/, Annals of Internal Medicine at http://www.annals.org/, and Epidemiology at http://www.epidem.com/). Information on the STROBE Initiative is available at www.strobe-statement.org.

**Supplemental References:**

S1. Makridakis, M., et al., Multiplexed MRM-based protein quantification of putative prognostic biomarkers for chronic kidney disease progression in plasma. Sci Rep, 2020. 10(1): p. 4815.

S2. Glorieux, G., et al., New insights in molecular mechanisms involved in chronic kidney disease using high-resolution plasma proteome analysis. Nephrol Dial Transplant, 2015. 30(11): p. 1842-52.

S3. Peters, K.E., et al., Identification of Novel Circulating Biomarkers Predicting Rapid Decline in Renal Function in Type 2 Diabetes: The Fremantle Diabetes Study Phase II. Diabetes Care, 2017. 40(11): p. 1548-1555.

S4. Manjunatha, S., et al., Functional and proteomic alterations of plasma high density lipoproteins in type 1 diabetes mellitus. Metabolism, 2016. 65(9): p. 1421-31.

S5. Sorensen-Zender, I., et al., Zinc-alpha2-glycoprotein in patients with acute and chronic kidney disease. BMC Nephrol, 2013. 14: p. 145.

S6. Pelletier, C.C., et al., The relationship between renal function and plasma concentration of the cachectic factor zinc-alpha2-glycoprotein (ZAG) in adult patients with chronic kidney disease. PLoS One, 2014. 9(7): p. e103475.

S7. Liabeuf, S., et al., Plasma beta-2 microglobulin is associated with cardiovascular disease in uremic patients. Kidney Int, 2012. 82(12): p. 1297-303.

S8. Puthiyottil, D., et al., Role of Urinary Beta 2 Microglobulin and Kidney Injury Molecule-1 in Predicting Kidney Function at One Year Following Acute Kidney Injury. Int J Nephrol Renovasc Dis, 2021. 14: p. 225-234.

S9. Xun, C., et al., Circulating RBP4 Increase and Its Diagnosis of Chronic Kidney Disease. Ann Clin Lab Sci, 2018. 48(2): p. 205-207.

S10. Frey, S.K., et al., Isoforms of retinol binding protein 4 (RBP4) are increased in chronic diseases of the kidney but not of the liver. Lipids Health Dis, 2008. 7: p. 29.

S11. Jain, S., V. Gautam, and S. Naseem, Acute-phase proteins: As diagnostic tool. J Pharm Bioallied Sci, 2011. 3(1): p. 118-27.

S12. Choi, J., et al., The co-chaperone DNAJC12 binds to Hsc70 and is upregulated by endoplasmic reticulum stress. Cell Stress Chaperones, 2014. 19(3): p. 439-46.

S13. Merchant, M.L., et al., Plasma kininogen and kininogen fragments are biomarkers of progressive renal decline in type 1 diabetes. Kidney Int, 2013. 83(6): p. 1177-84.

S14. Hong, Q., et al., LRG1 Promotes Diabetic Kidney Disease Progression by Enhancing TGF-beta-Induced Angiogenesis. J Am Soc Nephrol, 2019. 30(4): p. 546-562.

S15. Xiao, D., et al., Lumican promotes joint fibrosis through TGF-beta signaling. FEBS Open Bio, 2020. 10(11): p. 2478-2488.

S16. Gonzalez-Calero, L., et al., Urinary Kininogen-1 and Retinol binding protein-4 respond to Acute Kidney Injury: predictors of patient prognosis? Sci Rep, 2016. 6: p. 19667.

S17. Inker, L.A., et al., New Creatinine- and Cystatin C-Based Equations to Estimate GFR without Race. N Engl J Med, 2021. 385(19): p. 1737-1749.

S18. Vylet'al, P., et al., Alterations of uromodulin biology: a common denominator of the genetically heterogeneous FJHN/MCKD syndrome. Kidney Int, 2006. 70(6): p. 1155-69.

S19. Zivna, M., et al., Dominant renin gene mutations associated with early-onset hyperuricemia, anemia, and chronic kidney failure. Am J Hum Genet, 2009. 85(2): p. 204-13.

S20. Blumenstiel, B., et al., Development and Validation of a Mass Spectrometry-Based Assay for the Molecular Diagnosis of Mucin-1 Kidney Disease. J Mol Diagn, 2016. 18(4): p. 566-71.

S21. Zivna, M., et al., Noninvasive Immunohistochemical Diagnosis and Novel MUC1 Mutations Causing Autosomal Dominant Tubulointerstitial Kidney Disease. J Am Soc Nephrol, 2018. 29(9): p. 2418-2431.

S22. Murray, S.L., et al., Utility of Genomic Testing after Renal Biopsy. Am J Nephrol, 2020. 51(1): p. 43-53.

S23. Elhassan, E.A.E., et al., The utility of a genetic kidney disease clinic employing a broad range of genomic testing platforms: experience of the Irish Kidney Gene Project. J Nephrol, 2022. 35(6): p. 1655-1665.

S24. Hartmannová, H., et al., Acadian variant of Fanconi syndrome is caused by mitochondrial respiratory chain complex I deficiency due to a non-coding mutation in complex I assembly factor NDUFAF6. Hum Mol Genet, 2016. 25(18): p. 4062-4079.

S25. Chang, C.C., et al., Second-generation PLINK: rising to the challenge of larger and richer datasets. Gigascience, 2015. 4: p. 7.

S26. Gandini, R., et al., Structural basis for dolichylphosphate mannose biosynthesis. Nat Commun, 2017. 8(1): p. 120.

S27. Landmann, L., Deconvolution improves colocalization analysis of multiple fluorochromes in 3D confocal data sets more than filtering techniques. J Microsc, 2002. 208(Pt 2): p. 134-47.

S28. Manders, E.M.M., F.J. Verbeek, and J.A. Aten, Measurement of co-localization of objects in dual-colour confocal images. J Microsc, 1993. 169(3): p. 375-382.

S29. Vylet'al, P., et al., Plasma Mucin-1 (CA15-3) Levels in Autosomal Dominant Tubulointerstitial Kidney Disease due to MUC1 Mutations. Am J Nephrol, 2021. 52(5): p. 378-387.

S30. Seta, N., et al., Diagnostic value of Western blotting in carbohydrate-deficient glycoprotein syndrome. Clin Chim Acta, 1996. 254(2): p. 131-40.

S31. Darebna, P., et al., Changes in the expression of N- and O-glycopeptides in patients with colorectal cancer and hepatocellular carcinoma quantified by full-MS scan FT-ICR and multiple reaction monitoring. J Proteomics, 2017. 153: p. 44-52.

S32. Rappsilber, J., M. Mann, and Y. Ishihama, Protocol for micro-purification, enrichment, pre-fractionation and storage of peptides for proteomics using StageTips. Nat Protoc, 2007. 2(8): p. 1896-906.

S33. Tyanova, S., T. Temu, and J. Cox, The MaxQuant computational platform for mass spectrometry-based shotgun proteomics. Nat Protoc, 2016. 11(12): p. 2301-2319.

S34. Cox, J., et al., Andromeda: a peptide search engine integrated into the MaxQuant environment. J Proteome Res, 2011. 10(4): p. 1794-805.

S35. Cox, J., et al., Accurate proteome-wide label-free quantification by delayed normalization and maximal peptide ratio extraction, termed MaxLFQ. Mol Cell Proteomics, 2014. 13(9): p. 2513-26.

S36. Tyanova, S. and J. Cox, Perseus: A Bioinformatics Platform for Integrative Analysis of Proteomics Data in Cancer Research. Methods Mol Biol, 2018. 1711: p. 133-148.

S37. Perez-Riverol, Y., et al., The PRIDE database and related tools and resources in 2019: improving support for quantification data. Nucleic Acids Res, 2019. 47(D1): p. D442-D450.
